# Mapping actionable pathways and mutations in brain tumours using targeted RNA next generation sequencing

**DOI:** 10.1186/s40478-019-0826-z

**Published:** 2019-11-20

**Authors:** Krissie Lenting, Corina N. A. M. van den Heuvel, Anne van Ewijk, Duaa ElMelik, Remco de Boer, Elizabeth Tindall, Ge Wei, Benno Kusters, Maarten te Dorsthorst, Mark ter Laan, Martijn A. Huynen, William P. Leenders

**Affiliations:** 1 Department of Biochemistry, Radboud Institute for Molecular Life Sciences, Geert Grooteplein 26, 6525 GA Nijmegen, The Netherlands; 2 Center for Molecular and Biomolecular Informatics, Radboud Institute for Molecular Life Sciences, Geert Grooteplein 26, 6525 GA Nijmegen, The Netherlands; 3 Ignyta Inc, 4545 Towne Centre Court, San Diego, CA 92121 USA; 4 Department of Pathology, Radboud University Medical Center, Geert Grooteplein 10, 6525 GA Nijmegen, The Netherlands; 5 Department of Neurosurgery, Radboud University Medical Center, Geert Grooteplein 10, 6525 GA Nijmegen, The Netherlands

**Keywords:** Glioma, Pathways, Diagnostics, Prognostics, Targeted RNA-sequencing, Single molecule molecular inversion probes, Personalized

## Abstract

Many biology-based precision drugs are available that neutralize aberrant molecular pathways in cancer. Molecular heterogeneity and the lack of reliable companion diagnostic biomarkers for many drugs makes targeted treatment of cancer inaccurate for many individuals. Identifying actionable hyperactive biological pathways in individual cancers may improve this situation.

To achieve this we applied a novel targeted RNA next generation sequencing (t/RNA-NGS) technique to surgically obtained glioma tissues. The test combines mutation detection with analysis of biological pathway activities that are involved in tumour behavior in many cancer types (e.g. tyrosine kinase signaling, angiogenesis signaling, immune response, metabolism), via quantitative measurement of transcript levels and splice variants of hundreds of genes. We here present proof of concept that the technique, which uses molecular inversion probes, generates a histology-independent molecular diagnosis and identifies classifiers that are strongly associated with conventional histopathology diagnoses and even with patient prognosis. The test not only confirmed known glioma-associated molecular aberrations but also identified aberrant expression levels of actionable genes and mutations that have so far been considered not to be associated with glioma, opening up the possibility of drug repurposing for individual patients. Its cost-effectiveness makes t/RNA-NGS to an attractive instrument to aid oncologists in therapy decision making.

## Introduction

Many different cancer types share similar driver mutations. Examples are loss of function mutations in tumour suppressor genes (e.g. *p53* and *CDKN2A)* and DNA repair proteins*,* leading to genetic instability and loss of cell cycle control) [1–3], and activating mutations, amplifications or fusion events in proto-oncogenes [4, 5]. Other shared features are related to micro-environmental effectors, such as altered metabolism as a result of hypoxia [6, 7], induction of angiogenesis [8] and immune suppression [9].

Additionally, aberrations exist that are tumour-type specific. Examples are expression of hormone receptors in cancers of prostate, ovary and breast [10, 11]; mutations affecting metabolism (isocitrate dehydrogenase mutations [p.IDH1-R132H] in glioma and acute myeloid leukemia [12]); and mutations affecting the PI3K and MAPK pathway (PIK3CA, p.KRAS-G12/G13 mutations in adenocarcinomas [13], p.BRAF-V600E in melanoma [14]). Such specificity is however never absolute. As an example, *IDH*- and *BRAF* mutations are sporadically found in other cancers too [15–17]. Detection of such relatively rare, and therefore a priori unexpected mutations in individual patients could lead to repurposing of precision medicines in basket trials, in which precision drugs are administered to patients based on DNA profiling [18–21].

A number of actionable biological pathways in cancer involve the products of genes that are not mutated, but epigenetically regulated, for example by altered transcription factor availability, repressor activity or gene methylation, [22, 23]. Activity of such pathways cannot be directly inferred from DNA analyses. Whole genome methylation analysis has robust diagnostic power [24] but does not allow analysis of activity of biological pathways, involved in cancer development and progression. An example is angiogenesis, initiated by hypoxia-inducable factor (HIF-1α)- induced expression of an abundance of growth factors and followed by extensive crosstalk between tumour cells, tip- and stalk endothelial cells and pericytes [25, 26]. DNA analysis also does not provide information on post-transcriptional events. For example, expression of alternative splice variants of vascular endothelial growth factor (VEGF-A) has implications for the regulation of angiogenesis [27] and splice variants of receptor tyrosine kinases can lead to auto-active and oncogenic PI3K signaling (e.g. EGFR^VIII^ and MET^Δ7–8^ in glioma and MET^Δ14^ in lung cancer [28–31]).

A comprehensive overview of gene expression levels and alternative splice variants can be obtained with whole RNA next generation sequencing (w/RNA-NGS), provided sufficient coverage to detect alternative exon-exon boundaries. w/RNA-NGS is increasingly performed in a research setting but is cost-wise still not suitable for implementation in routine patient care. There is therefore a huge need for novel and cost-effective methods to obtain clinically actionable and reliable information for individual patients, to be able to implement personalized treatment approaches.

Due to its low incidence (6 per 100,000) and high molecular heterogeneity [32], glioma is a difficult tumour type to organize clinical trials with, although the molecular underpinnings of gliomagenesis and glioma progression are relatively well established [12]. In the absence of alternatives, treatment of its most malignant form, glioblastoma, is still confined to palliative surgery, followed by chemotherapy with temozolomide (TMZ) and radiotherapy [33] which extents median life expectancy with only few months. Surgical cure for this tumour type is not possible due to its diffuse infiltrative nature [34]. Glioma is therefore one of the most challenging tumours for which new treatment strategies are urgently needed.

We here analyzed test and validation cohorts of in total 103 surgically derived brain tumours with quantitative targeted RNA next generation sequencing (t/RNA-NGS) [35–38]. The technique uses single molecule molecular inversion probes (smMIPs) and sensitively and quantitatively measures expression levels of and mutations in actionable genes. We show that t/RNA-NGS provides a histology-independent molecular diagnosis and identifies classifier transcripts that are closely associated with histopathological diagnosis and prognosis. By measuring hyperactivity of cancer-related pathways the test may also stratify individual patients for treatment with appropriate medicine.

## Materials and methods

### Patients

The study described here was performed with brain tumour tissue from newly diagnosed patients who were operated for a glioma between 2013 and 2018 (*n* = 103). The cohort was separated in a training cohort (*n* = 75, tumours that were operated between 2013 and 2017) and an independent validation set of 28 tumours (operated in 2017 and 2018). Researchers were blinded to histopathology and clinical outcome. The study protocol was approved by the Ethical Committee for Human Experimentation of the Radboudumc. All patients signed informed consent. Directly after surgery, tissue samples were snap frozen in liquid nitrogen and stored at -80 °C until further processing. In retrospect, patient characteristics were extracted from Radboudumc electronic patient files (EPIC) and documented in the electronic data capture system CASTOR. Histopathology and molecular diagnoses were extracted from the Dutch Pathology archive PALGA and summarized in Table 1.

**Table 1:** Summary of characteristics of glioma samples

Sample	Sex	Age (at sugery)	Histological type	Grade	Mutation status	% tumor cells
*TRAINING COHORT*						
13-01	F	28	Ependymoma	2	IDHwt	*
13-02	M	40	Astrocytoma	3	IDH1-R132H	70
13-03	M	58	Oligodendroglioma	3	IDH1-R132H	70
13-04	V	62	Glioblastoma	4	IDHwt	60
13-05	M	56	Metastasis	**	IDHwt	*
13-06	M	53	Oligodendroglioma	3	IDH2-R172K	60
13-07	F	20	Variant glioma	2	IDHwt	*
13-08	M	67	Glioblastoma	4	IDHwt	70
13-09	V	58	Glioblastoma	4	IDHwt	70
13-10	M	45	Oligodendroglioma	3	IDH1-R132H	65
13-11	V	67	Glioblastoma	4	IDHwt	70
13-13	M	52	Glioblastoma	4	IDH1-V178I	70
13-14	F	64	Glioblastoma	4	IDHwt	70
13-15	V	44	Oligodendroglioma	3	IDH1-R132H	50
13-16	M	60	Glioblastoma	4	IDHwt	70
13-17	M	45	Oligodendroglioma	2	IDH1-R132H	50
13-18	V	49	Oligodendroglioma	3	IDH1-R132H	50
14-01	V	52	Glioblastoma	4	IDHwt	80
14-02	M	43	Oligodendroglioma	2	IDH1-R132H	50
14-03	V	62	Glioblastoma	4	IDHwt	70
14-04	M	72	Glioblastoma	4	IDHwt	60
14-05	M	21	Astrocytoma	2	IDH1-R132H	70
14-06	M	43	Oligodendroglioma	3	IDH1-R132H	50
14-07	M	65	Oligodendroglioma	3	IDH1-R132H	50
14-08	M	50	Astrocytoma	3	IDH1-R132H	50
14-09	V	43	Astrocytoma	3	IDH1-R132H	60
14-10	V	45	Glioblastoma	4	IDH1-R132H	50
14-11	M	50	Glioblastoma	4	IDHwt	60
14-12	M	59	Oligodendroglioma	3	IDH1-R132H	50
14-13	M	39	Pleomorphous xanthoastrocytoma	3	IDHwt, BRAF-V600E	70
15-01	M	66	Glioblastoma	4	IDHwt	50
15-02	V	61	Glioblastoma	4	IDHwt	70
15-03	V	76	Glioblastoma	4	IDHwt	40
15-04	V	59	Glioblastoma	4	IDHwt	40
15-05	M	31	Astrocytoma	3	IDH1-R132H	70
15-06	V	49	Astrocytoma	3	IDH1-R132H/V178I	70
15-07	M	63	Glioblastoma	4	IDH1-V178I	65
15-08	M	55	Astrocytoma	2	IDH1_R132H	60
15-09	M	70	Glioblastoma	4	IDHwt	70
15-10	V	68	Oligodendroglioma	3	IDH1-R132H	70
15-11	M	33	LPD	**	IDHwt	*
15-12	M	46	Glioblastoma	4	IDHwt	70
15-13	V	78	Glioblastoma	4	IDHwt	80
15-14	M	79	Glioblastoma	4	IDHwt	70
15-15	V	58	Glioblastoma	4	IDHwt	70
15-16	M	25	Astrocytoma	2	IDH1-R132H/V178I	50
15-17	M	68	Glioblastoma	4	IDHwt	60
15-18	V	64	Glioblastoma	4	IDHwt	70
16-01	M	61	Glioblastoma	4	IDHwt	70
16-02	M	47	Glioblastoma	4	IDHwt	70
16-03	V	46	Astrocytoma	3	IDH1-R132H	25
16-04	M	59	Oligodendroglioma	3	IDH1-R132H	60
16-05	M	51	Glioblastoma	4	IDHwt	50
16-06	V	*	Astrocytoma	*	IDH1-R132H	50
16-07	M	74	Glioblastoma	4	IDH1-Y183C	60
16-08	F	68	Glioblastoma	4	IDHwt	50
16-09	V	49	Glioblastoma	4	IDHwt	70
16-10	M	*	Astrocytoma	*	IDH1-R132H	60
16-11	M	67	Glioblastoma	4	IDHwt	50
16-12	M	23	Astrocytoma	3	IDH1-R132H	60
16-13	M	60	Glioblastoma	4	IDHwt	70
16-14	V	60	Oligodendroglioma	3	IDH2-R172M	70
16-15	V	61	Oligodendroglioma	3	IDH1-R132H	70
16-16	M	58	Glioblastoma	4	IDH1-V178I	40
16-17	V	18	Oligodendroglioma	2	IDH2-R172K	40
16-18	*	30	Oligodendroglioma	3	IDH2-R172W	70
16-19	M	48	Glioblastoma	4	IDHwt	70
17-01	M	58	Oligodendroglioma	2	IDH1-R132H	50
17-02	M	40	Astrocytoma	3	IDH1-R132H/V178I	70
17-03	V	76	Glioblastoma	4	IDHwt	65
17-04	M	42	Oligodendroglioma	3	IDH1-R132H	70
17-05	M	59	Glioblastoma	4	IDHwt	70
17-06	M	65	Glioblastoma	4	IDHwt	70
17-07	M	63	Glioblastoma	4	IDHwt	70
17-08	F	26	DNET	1	IDHwt	*
*VALIDATION COHORT*						
17-09	F	67	Glioblastoma	4	IDHwt	*
17-10	F	67	Astrocytoma	2	IDH1-R132H	*
17-11	M	67		4	IDHwt	*
17-12	*	*			IDH1-V178I	*
17-13	F	59	Glioblastoma	4	IDHwt	75
17-14	M	46	Oligodendroglioma	2	IDH1-R132H	*
17-16	M	56	Astrocytoma	3	IDH1-R132H	*
18-01	F	48	oligodendroglioma	2	IDH1-R132H	60
18-02A	F	39	Astrocytoma	2	IDHwt	*
18-02B	F	44	Astrocytoma	2	IDHwt	*
18-04	M	65	Glioblastoma	4	IDHwt	65
18-05	M	19	Astrocytoma	3	IDHwt	45
18-06	M	48	Glioblastoma	4	IDHwt	70
18-07	M	76	Glioblastoma	4	IDHwt	*
18-08	M	69	unknown		IDHwt	45
18-09	M	39	Astrocytoma	3	IDH1-R132H	80
18-10	M	53	Glioblastoma	4	IDHwt	80
18-12	M	51	oligodendroglioma	2	IDH1-R132H	few
18-13	M	42	Pleomorphous xanthoastrocyoma	3	BRAF-V600E	*
18-14	M	53	Other		IDHwt	40
18-15	M	67	Glioblastoma	4	IDHwt	65
18-16	M	76	Glioblastoma	4	n.d.	70
18-17	F	45	Oligodendroglioma	2	IDH1-R132H	70
18-18	F	72	Glioblastoma	4	n.d.	75
18-19	M	55	Glioblastoma	4		80
18-20	F	54	Glioblastoma	4	IDH1-V178I	80
18-21	M	53	Other	4	IDHwt	75
18-23						80

Histological type, WHO grade, and percentage tumor cells were confirmed by a trained neuropathologist (B.K.). IDH-mutational status was derived from the t/RNA-seq data. All IDH1-R132H/IDH2 mutations were validated by routine diagnostic genetic analysis with the ‘Radboud Cancer Hotspot Gene panel’.

Abbreviations: *DNET* Dysembryoplastic neuroepithelial tumor, *F* Female, *IDH* Isocitrate dehydrogenase, *LPD* Lymphoproliferative disorder, *M* Male, *IDHWT* Wild-type, *WHO* World Health Organization. * data not available; ** non-glioma, no WHO-grade

### RNA preparation and cDNA synthesis

Cryosections of 10 μm were cut for RNA isolation using TRIzol (ThermoFisher Scientific, Waltham, MA). For every sample a 4 μm serial section was stained with H&E to estimate percentage tumour area by an experienced neuropathologist (BK). RNA was reverse transcribed into cDNA using random hexamer primers and Superscript II (Invitrogen, CA) according to standard protocols. In parallel, tissue was processed to formalin-fixed paraffin-embedded (FFPE) tissue blocks for routine diagnosis. Samples from 2014 onwards were also subjected to genetic analysis using targeted DNA next generation sequencing [18].

### T/RNA-NGS with smMIPs

All enzymes were from NEB (Ipswich, MA) unless stated otherwise. The procedure of smMIP-based targeted RNA Next Generation Sequencing (t/RNA-NGS) to detect expression of metabolic genes has been described before [35, 37–39]. SmMIPs were designed by an adjusted version of the MIPgen algorithm [40] and were ordered from Integrated DNA Technologies (Leuven, Belgium). The set of smMIP probes used in [35] was expanded with probes for detection of regions of interest (ROI) in novel transcripts of interest that play a role in a wide variety of cancer types. Furthermore, smMIP probes were included for specific detection of splice variants by placing extension and ligation probes on neighboring exons, e.g. probes on exons 1/8 of Epidermal Growth Factor Receptor (EGFR) for detection of EGFR^vIII^ [37]. Probes for detection of Vascular Endothelial Growth Factor (VEGF) isoforms 121, 165 and 189 were designed with extension and ligation probes in exons 5/8, 5/7 and 5/6, respectively. Additional smMIPs were included that were directed against shared exons of transcript variants. For transcripts in which relevant mutations can be expected, smMIPs were selected with extension and ligation probes flanking the respective ROI. At least 5 smMIPs were used for each transcript of interest, evenly distributed along the transcript. cDNA was subjected to smMIP capture as described in [35, 36]. In short, a mixture of 936 phophorylated smMIPs was hybridized to 15–50 ng of cDNA. After overnight hybridization and enzymatic gap-filling (KlenTaq polymerase, Epicentre, Madison, WI) and ligation (Ampligase, Epicentre) circular smMIPs are formed, the number of which is linearly related to the number of RNA molecules present in the original sample. After treatment with exonuclease to remove cDNA and linear smMIPs, the libraries of circular smMIPs were subjected to PCR with a unique barcoded primer set per sample. The PCR products of the expected length of 266 bp were purified with Ampure beads (Beckmann Coulter Genomics, High Wycombe, UK) and quantified on a TapeStation 2200 (Agilent Technologies, Santa Clara, CA). PCR libraries were sequenced on the Illumina Nextseq platform (Illumina, San Diego, CA) at the Radboudumc sequencing facility (output 2 × 151 bases). The barcode in the PCR primers allows for pooling of multiple samples into one sequencing library, followed by demultiplexing of reads, generating for all individual samples a FASTQ file.

### Data processing

For each patient sample, all reads in the FASTQ file were mapped against reference transcripts (UCSC human genome assembly hg19) using the SeqNext module of JSI SequencePilot version 4.2.2 build 502 (JSI Medical Systems, Ettenheim, Germany). In all smMIP molecules a unique random 8 N sequence is included (unique molecular identifier or UMI), allowing to assign all identical sequencing reads to one originating circularized (unique) smMIP. This excludes PCR amplification bias and makes the assay quantitative. Number of unique reads for each individual smMIP in a sample were normalized to the total of unique reads in that sample and expressed as fragment per million (FPM). Mean FPM values from all smMIPs covering different ROIs in the same transcript were considered to represent gene expression levels in the tumour sample. Using the base calling algorithm of SeqNext a list of single nucleotide variations and insertions and deletions was generated for each sample. An in–house developed Python script (version 3.7) allowed direct coupling of gene expression values and mutation status of individual cancer samples.

### Data analysis and statistics

Unsupervised hierarchical cluster analysis with the gene expression data was performed using R programming (version 3.4.3). Mean FPM values were log-transformed (after addition of 0.01 to prevent log0 transformation),. Manhattan distance between gene expression profiles were calculated using the group average method for agglomerative clustering (Unweighted Pair Group Method with Arithmetic Mean) [41]. Other packages were heatmap. Plus (http://www.sciviews.org/SciViews-R.) and plyr: http://www.jstatsoft.org/v40/i01/. The Wilcoxon Mann-Whitney U test was used to find differentially expressed genes between clusters. Associations of mutations with clusters were calculated using Fisher’s exact test. Multiple testing corrections were done using Benjamini Hochberg (FDR < 0.05). Kaplan-Meier survival curves of 75 brain cancer patients in the test cohort were generated using Graphpad Prism, version 5.03. Patients who were still alive at the date of analysis or were lost to follow-up, were censored in the survival data. *P*-values were calculated using the Log-Rank test. All *p*-values are indicated as *(*p* < 0.05), ** (*p* < 0.01), *** (*p* < 0.001), **** (*p* < 0.0001), unless specified otherwise. Survival analysis of the 28 patients in the validation cohort could not be performed due to short follow-up time.

### Immunohistochemistry

Immunohistochemistry (IHC) was performed on 4 μm sections of FFPE tissue or on frozen sections, adjacent to those used for t/RNA-NGS. Frozen sections were air dried and fixed with 4% paraformaldehyde (PFA) solution for 20 min at room temperature before staining. After appropriate epitope retrieval (for FFPE sections), antibodies rabbit-anti-Prostate-Specific Membrane Antigen (PSMA) (Abcam; ab133579), rabbit-anti-Carbonic Anhydrase 12 (CA12) (Sigma Life Sciences; HPA008773), mouse-anti-androgen receptor (AR) (Santa Cruz; sc-7305), rabbit-anti-MET (Cell Signaling Technologies; #8198), and rabbit-anti-EGFR (Cell Signaling Technologies; #4267) were used. Sections were incubated with primary antibody in normal antibody diluent (Immunologic, Duiven, The Netherlands) overnight at 4 °C. Primary antibody detection was done using BrightVision polyHRP-anti-rabbit IgG (Immunologic, Duiven, The Netherlands), or BrightVision polyHRP-anti-mouse/rabbit/rat IgG (Immunologic) for AR staining. Sections were counterstained with haematoxylin and mounted with Quick-D mounting medium (Klinipath, Duiven, The Netherlands). As control staining, secondary antibody-only stainings were performed.

### Whole transcriptome RNA-NGS (w/RNA-NGS)

Total RNA was isolated using the Qiagen AllPrep DNA/RNA Mini Kit following the manufacturer’s protocol for animal tissues (Qiagen, Hilden, Germany). RNA sequencing libraries were prepared from 16 gliomas using the KAPA RNA HyperPrep Kit with RiboErase (HMR) (KAPA Biosystems, Wilmington, MA) following the manufacturer’s protocol. Briefly, 100 ng of total RNA was used to generate libraries, which were fragmented for 8 min at 94 °C. Adapter stock concentration used was 750 nM and libraries were amplified for 11 cycles. Duplex “Y” adapter sequences with molecular barcodes were generated by IDT (Integrated DNA Technologies, Skokie, Illinois). Final libraries were quantified on a High Sensitivity Bioanalyzer chip (Agilent, Santa Clara, CA) and sequenced at 1.4pM with 10% PhiX on the Illumina NextSeq 550 Sequencer (Illumina, San Diego, CA). Raw FASTQ files were mapped to the human genome (hg19) with STAR (v2.5.3) aligner [42]. Mapped reads were filtered and deduplicated using sambamba v0.6.6 and feature quantification was performed using featureCounts v1.5.0-p1 against the RefSeq database (downloaded from the UCSC genome browser on 01/05/2017). Gene and exon-level fragments per kilobase per million mapped reads (FPKMs) were calculated using a custom python script.

## Results

### T/RNA-NGS profiles have prognostic value

**Fig. 1:**
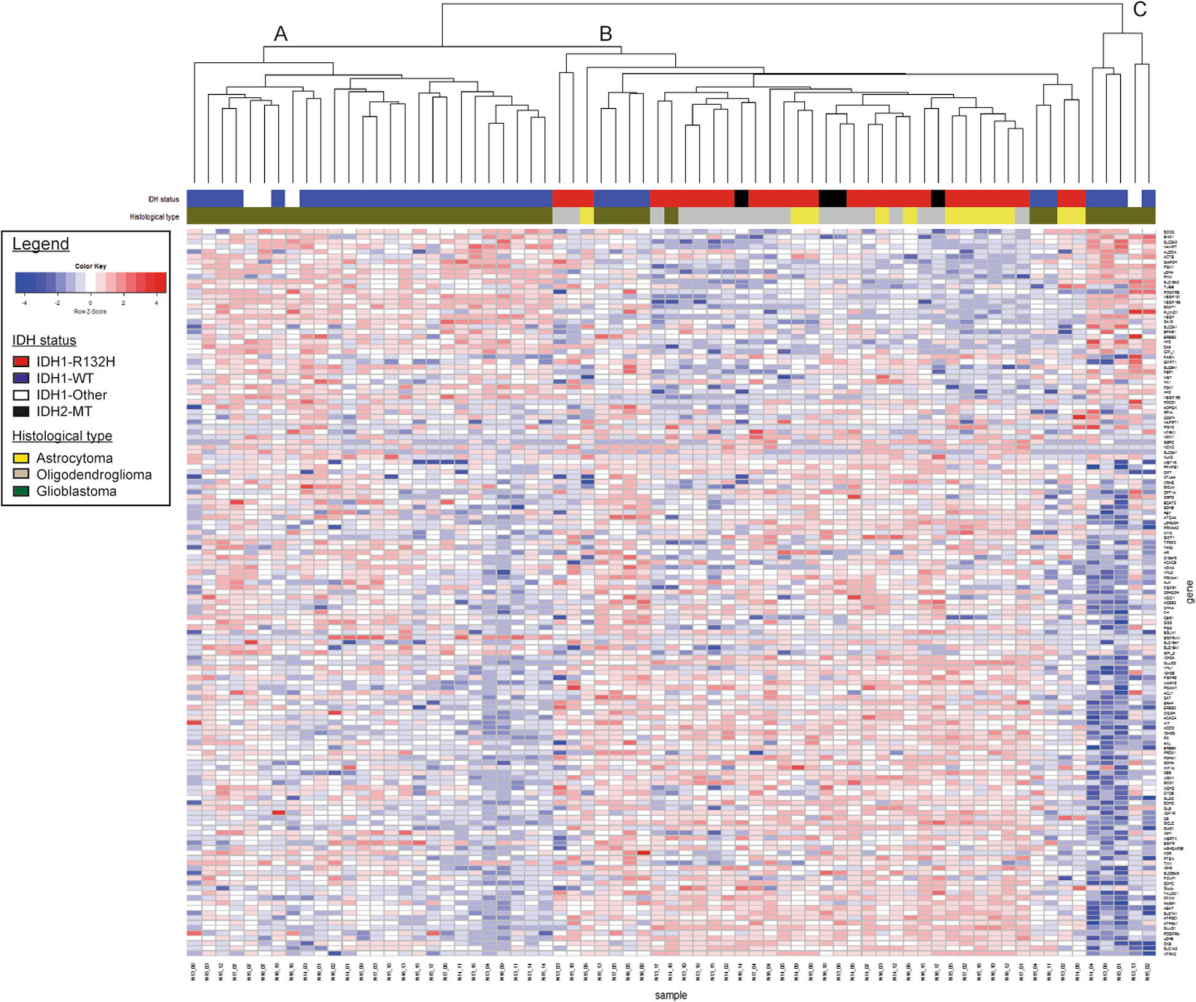
Agglomerative clustering of t/RNA-NGS profiles of 69 grade II-IV gliomas. Clustering was based on expression levels of 145 genes of interest. Gene expression levels (in FPM) were transformed to a z-score for each individual transcript. After generating the dendrogram and heatmap using the average clustering method in R, histopathology results and mutation status were added in retrospect (legend and upper annotation bars). Cluster A and C strongly correlate with a wild-type IDH status, while cluster B is strongly associated with the IDH1^R132H^ mutation (see also Additional file 4: Table S3). IDH2 mutations (R172K, R172M, and R172W) are annotated as IDH2-MT (black in the annotation bar) and all coclustered with the IDH1^R132H^ mutation. IDH1-other refer to variants other than the IDH1^R132H^ (see Table 1)

To investigate the prognostic value of t/RNA-NGS, we profiled a training set of 75 brain tumours, including 69 grade II-IV diffuse gliomas and 6 brain lesions that upon routine histopathology were diagnosed as an ependymoma (*n* = 1), one dysembryoplastic neuroepithelial tumour (DNET), one pleomorphic xanthoastrocytoma, a variant glioma, a brain metastasis of lung adenocarcinoma and a lymphoproliferative disorder (LPD) (Table 1). Annotated unique smMIP counts for each tumour sample ranged from 275,000 to 1,111,000 (not shown). Hierarchical unsupervised agglomerative clustering of the gene expression data of the grade II-IV gliomas (excluding the 6 rare cancers) resulted in 3 main clusters A, B and C, comprising of 26, 38 and 5 tumours, respectively (Fig. 1).

In a next step we performed a Wilcoxon Mann-Whitney U test to find genes that were differentially expressed between clusters A and B; A and C; and B and C. A total of 83 genes were differentially expressed between clusters A and B with *p* < 0.05 and False Discovery Rate (FDR) < 0.05 (Additional file 3: S2a). Among these were transcripts encoding transporters and enzymes involved in metabolism, as described before [39]. Membrane receptor tyrosine kinases (RTKs) NTRK2, ERBB3, ERBB4 were higher expressed in cluster B with average fold changes (FC) of 3.5, 4.8 and 5.2 respectively, while cluster A was characterized by significantly higher expression levels of EGFR^vIII^ (FC = 250) and VEGF-A isoforms VEGF-A_121_, VEGF-A_165_ and VEGF-A_189_ (all with FC > 11, Additional file 2: Table S1a). Between cluster B and C 69 genes were differentially expressed (Additional file 2: Table S1b). Cluster A and C significantly differed with respect to expression levels of 9 genes (Additional file 2: Table S1c). The functional significance of these differences were not subject of further investigation in this study.

**Fig. 2:**
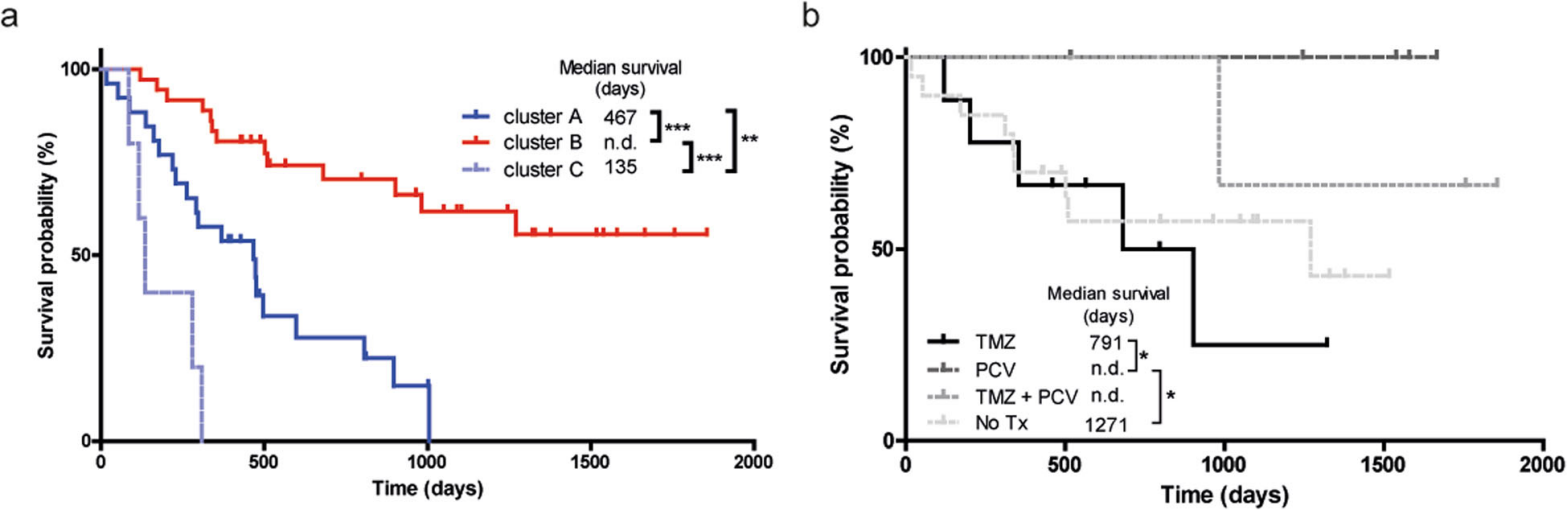
Kaplan-Meier analyses of unsupervised clusters. **a** For patients in clusters A (*n* = 26) and C (*n* = 5) median survival was 467 days and 135 days, respectively. For cluster B (*n* = 38) the follow-up period was too short to determine median survival. Survival of patients in cluster B was significantly better than of clusters A and C (both *p* < 0.0001). Survival in cluster A was significantly better than C (*P* = 0.008). **b** Oligodendroglioma patients in cluster B (diagnosed according to WHO 2016 classification, and treated with PCV) performed better than astrocytoma patients in this cluster (all treated with TMZ) (*p* = 0.04). Two patients (N16–06 and N16–10) were excluded from analysis because no survival data was present

We then coupled the profiles to survival data. As shown in Fig. 2a, cluster B gene expression profiles of gliomas were associated with good prognosis (median survival, defined as time between surgery and death, > 6 years; exact value could not be calculated for available follow-up time) whereas gene expression profiles in cluster A and C were associated with median survival of 467 and 135 days, respectively. Results were highly significant between clusters A and B (*p* < 0.0001), A and C (*p* = 0.0078), and C and B (p < 0.0001). A Kaplan-Meier curve with survival data including also the non-glioma patients is presented in Additional file 1: Figure SI.

### Mutation analysis

We next investigated whether we could identify nucleotide variations that significantly associate with the subgroups. To this end we included all identified sequence variants of potential clinical significance with a coverage of > 10% of the unique reads. This analysis revealed that the well known hotspot mutation in the metabolic enzyme isocitrate dehydrogenase 1 (p.IDH1-R132H) [12] was significantly associated with cluster B (*p* < 3 × 10^− 5^ when compared to cluster A, see Additional file 3: Table S2). Additionally a duplication in succinate dehydrogenase A (SDHA) of unknown significance was strongly associated with group B (*p* = 3 × 10^− 5^). The test also identified known oncogenic mutations in mitochondrial IDH2 in four patients (two p.R172K, one p.R172C and one p.R172M mutation, Table 1). Gene expression profiles from these gliomas co-clustered with those of the p.IDH1-R132H gliomas in cluster B (Fig. 1, black in IDH-status annotation bar). All IDH1^R132^ and IDH2^R172^ mutations were in retrospect confirmed by routine genetic analyses (not shown). As described before, also other variants of IDH1 (p.V178I, p.Y183C) were identified by the assay [39] (Table 1).

We then analyzed the profiles in relation to survival with treatment and histopathology as additional parameters. Retrospective analysis of histopathology and clinical follow-up data revealed that all patients in clusters A and C received temozolomide (TMZ) and/or radiotherapy upon signs of tumour progression after surgery, suggesting that the differences in survival were not related to treatment. We therefore concentrated on patients in cluster B who were treated with TMZ (*n* = 9, astrocytomas), procarbazine/lomustine/vincristine (PCV) chemotherapy (*n* = 6, all 1p/19q codeleted oligodendrogliomas), both adjuvant therapies (*n* = 3), or did not receive additional treatment (*n* = 19). Of 2 patients adjuvant therapy status was unknown (*n* = 2). As shown in Fig. 2b, outcome for oligodendroglioma patients treated with PCV was better than for astrocytoma patients treated with TMZ (*p* = 0.04).

Whereas group B was dominated by IDH-mutated gliomas, this group also contained 6 IDH wild-type glioblastomas. Comparison of gene expression profiles of these gliomas with those of cluster A revealed that expression levels of all VEGF isoforms were significantly lower in the group B *IDH*^*wt*^ gliomas (Additional file 4: Table S3). This suggests that these 6 tumours grouped with the *IDH*^*mut*^ gliomas based on the lack of an angiogenic response.

### T/RNA-NGS based molecular diagnosis

**Fig. 3:**
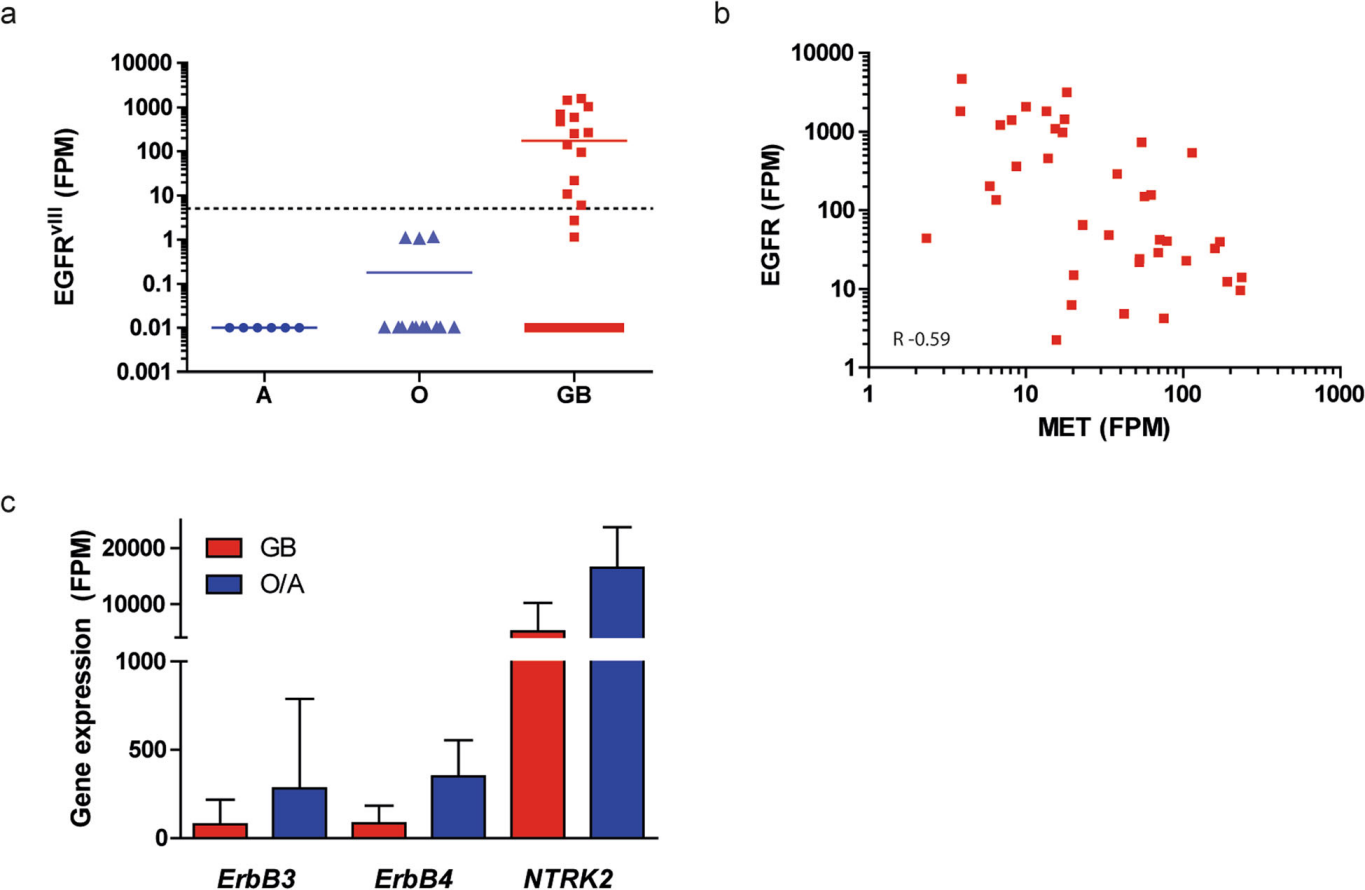
Actionable gene expression in grade II/III astrocytomas, oligodendrogliomas and glioblastoma. **a** EGFR^vIII^ was detected at significant levels in 30% of glioblastomas (GB, red) but not in grade II/III astrocytomas (A) and oligodendrogliomas (O) (note the log scale of the Y-axis, expression < 5 FPM was considered EGFR^vIII^-negative). **b** Wild-type EGFR expression is inversely correlated with expression of MET in glioblastoma (Spearman R = − 0.59; p < 0.0001). **c** Mean expression of *ErbB3, ErbB4*, and *NTRK2* in GB (red) and grade II/III O/A (blue). Error bars represent the standard deviation

To investigate whether t/RNA-NGS profiles are associated with histopathology diagnoses, we performed a group-based analysis according to the WHO 2016 grading system [43] and compared grade II/III astrocytomas (*n* = 12), grade II/III oligodendrogliomas (*n* = 19) and glioblastomas (*n* = 38). Wilcoxon Mann-Whitney U tests identified 79 genes that were differentially expressed between diffuse grade II/III oligodendrogliomas and glioblastomas (Additional file 5: Table S4a). In the top 5 of differentially expressed genes were *LDHA* and *BCAT1*, genes that are known to be hypermethylated in low grade, *IDH*^*mut*^ gliomas [36, 44]. Expression levels of 50 genes were significantly different between grade II/III astrocytomas and glioblastomas (Additional file 5: Table S4b), whereas expression levels of only 1 gene (*ALK*) differed significantly between astrocytomas and oligodendrogliomas (Additional file 5: Table S4c). As expected, both oligodendrogliomas and astrocytomas were distinguished from glioblastomas by the p.IDH1-R132H mutation. EGFR^vIII^ was expressed in 30% of glioblastomas (*n* = 13; mean FPM = 550, range 6–1450) (Fig.3a) and never in grade II/III gliomas, in good agreement with literature [45]. Expression of wild-type EGFR was found among all gliomas, but was more prominent in glioblastomas than in grade II/III gliomas (mean FPM = 612 vs. 126; *p* = 0.0061). Levels of EGFR and MET expression in glioblastomas were inversely correlated (Spearman R = -0.59, *p* < 0.0001; Fig. 3b). Not surprising given the strong association of IDH1 mutations and grade II/III gliomas with cluster B of Fig. 1, the supervised histopathology-based analysis again identified ErbB3, ErbB4 and TrkB (the product of *NTRK2*) as potentially targetable proteins in grade II/III gliomas (Fig. 3c).

### Carbonic anhydrase 12 is a poor prognostic factor

**Fig. 4:**
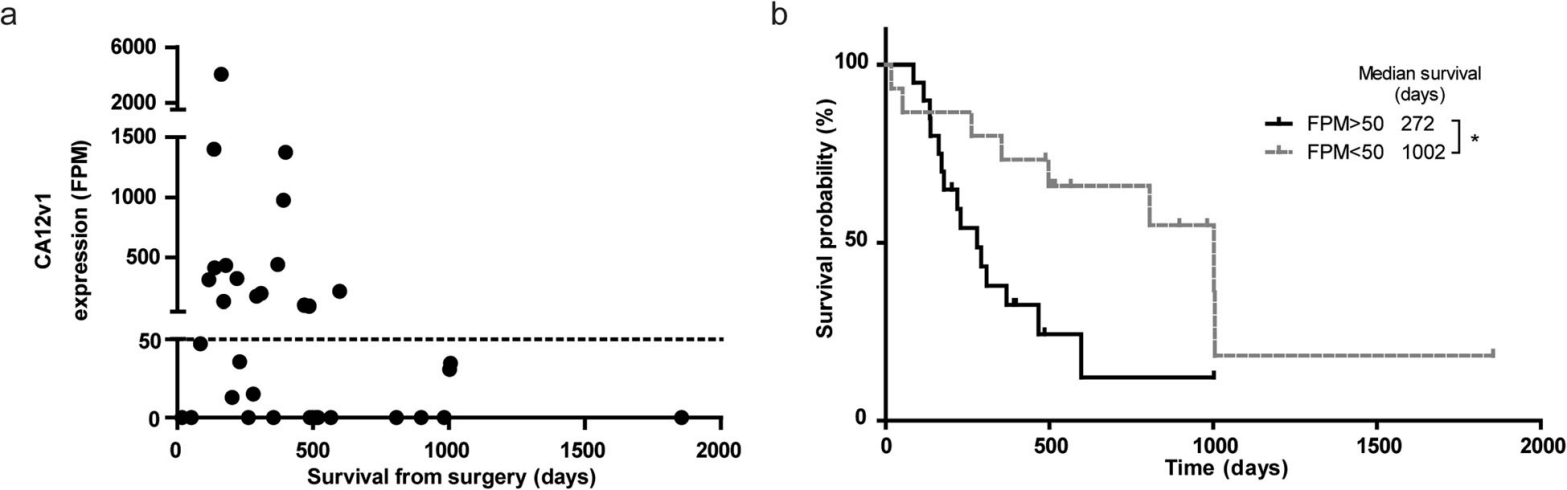
Expression of CA12 is a poor prognostic factor. **a** CA12v1 expression levels in the positive patient samples included in panel A. Expression > 50 FPM is a poor prognostic survival marker. **b** Kaplan-Meier curve of IDH^WT^ gliomas, grouped based on CA12v1 expression

To investigate whether prognostic factors other than *IDH1*-mutations can be identified by t/RNA-NGS, we performed subgroup analysis on tumour profiles from *IDH*^*wt*^ patients with survival < 14 months and > 14 months (the median overall survival time of this group of patients). A Fisher’s exact test on the sequence variations in these groups identified two different splice isoforms of CA12: a full-length variant 1 (CA12v1), and a variant 2 (CA12v2) that lacks exon 9. Expression levels of CA12v1 were associated with poor survival (Fig. 4a). Detection of this variant was based on one smMIP with both exon 8–9 and exon 8–10 boundaries in its ROI. This smMIP can detect both isoforms due to the small size of exon 9 of 33 nucleotides. Interestingly, CA12v1 was never detected in *IDH*^*mut*^ gliomas. CA12v1 expression values higher than 50 FPM translated in poor prognosis as shown by Kaplan-Meier analysis (272 vs.1002 days from surgery to death, *p* = 0.0137) (Fig. 4b).

### Validation of t/RNA-NGS data

**Fig. 5:**
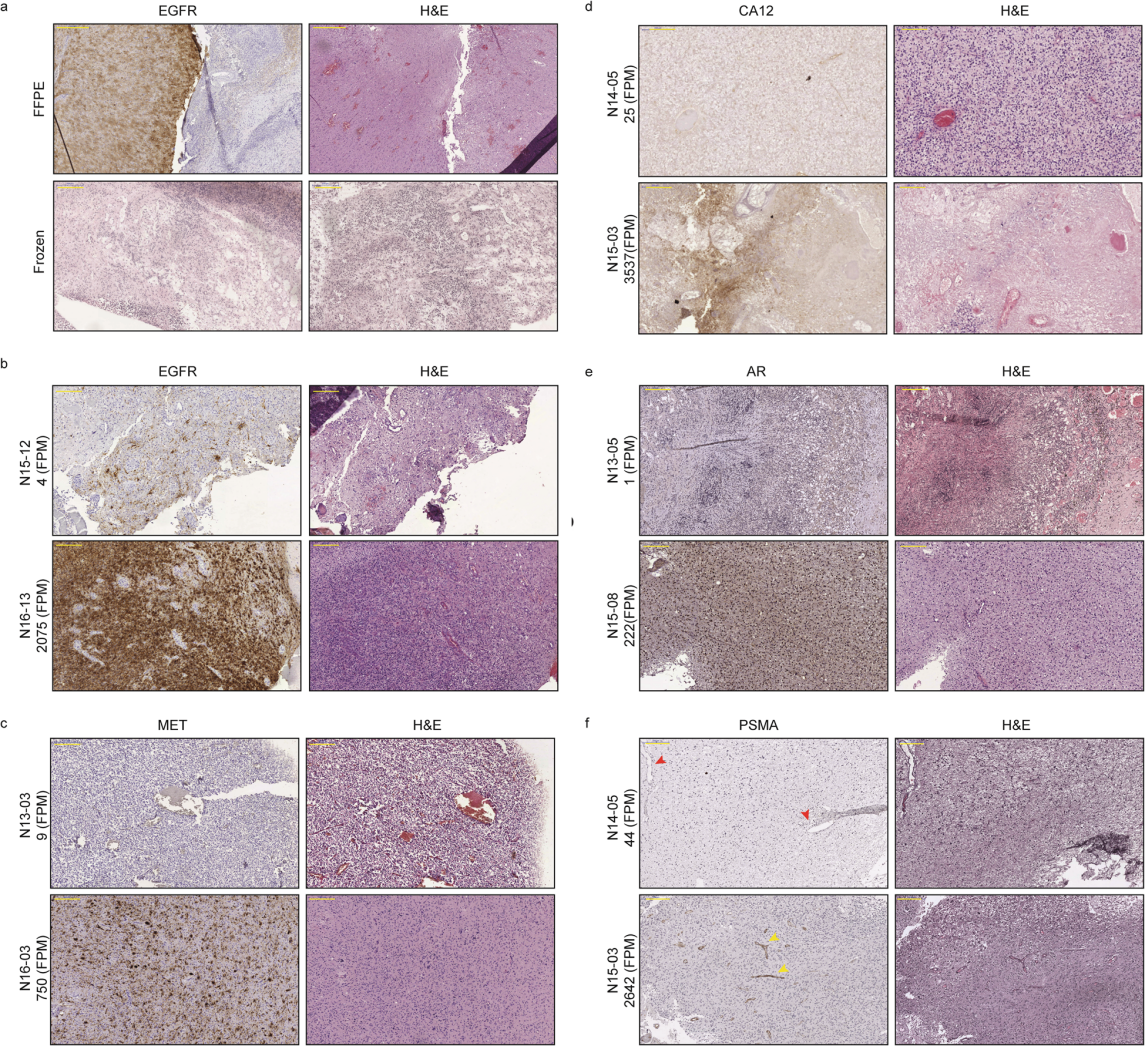
Immunohistochemical staining of EGFR, MET, CA12, AR, and PSMA. **a**/**b** EGFR staining of tumour N15–12 (negative in the smMIP assay) was negative in the frozen section, but partly positive in the FFPE section, indicating intratumoural heterogeneity of EGFR expression. High EGFR gene expression in tumour N16–13 was consistent with protein expression. IHC staining for MET (**c**), CA12 (**d**), and AR (**e**) of tumour samples with low (upper panels) and high (lower panels) expression levels of MET, CA12 and AR respectively in the t/RNA-NGS assay. Expression levels in FPM are stated with the sample name. **f** Staining for PSMA showed endothelial staining in gliomas with high PSMA FPM values, as indicated with the yellow arrowheads in the lower panel. In gliomas with low expression (N14–05), no staining was observed (red arrowheads in upper panel point at blood vessels). Original magnification for upper panel in A, 5x. For lower panel of A and other panels, magnification is 10x. Yellow annotated bar for 5 × 500 μm, for 10 × 200 μm

We have previously shown that the quality of t/RNA-NGS data correlates well with w/RNA-NGS [35]. All IDH1 and IDH2 mutations that were found with regular molecular screening, were verified by t/RNA-NGS [36]. Interestingly, the broad set-up of our t/RNA-NGS assay allowed the frequent detection of androgen receptor (AR) in gliomas at relatively high levels. To confirm and validate these and other findings on the proteome level we performed immunohistochemical analyses for EGFR, MET, CA12 and AR on a number of tumours with low or high FPM values for the corresponding genes. Transcript levels for EGFR, MET, CA12, and AR correlated with protein levels (Fig. 5). In one case we found that EGFR-transcript levels from frozen tumour tissue did not match with protein levels in FFPE blocks. This discrepancy could be attributed to tumour heterogeneity since the FFPE block contained both EGFR-positive and negative areas (Fig. 5a).

We also detected prominent expression of *FOLH1*, the gene encoding prostate specific membrane antigen (PSMA), in approximately 70% of the gliomas. To validate this finding we performed IHC analysis for PSMA on a number of tumours with either low or high *FOLH1* FPM values. IHC revealed expression of PSMA on the blood vessels of tumours with high *FOLH1* FPM values (Fig. 5f).

### Actionable markers in individual brain cancers

**Fig. 6:**
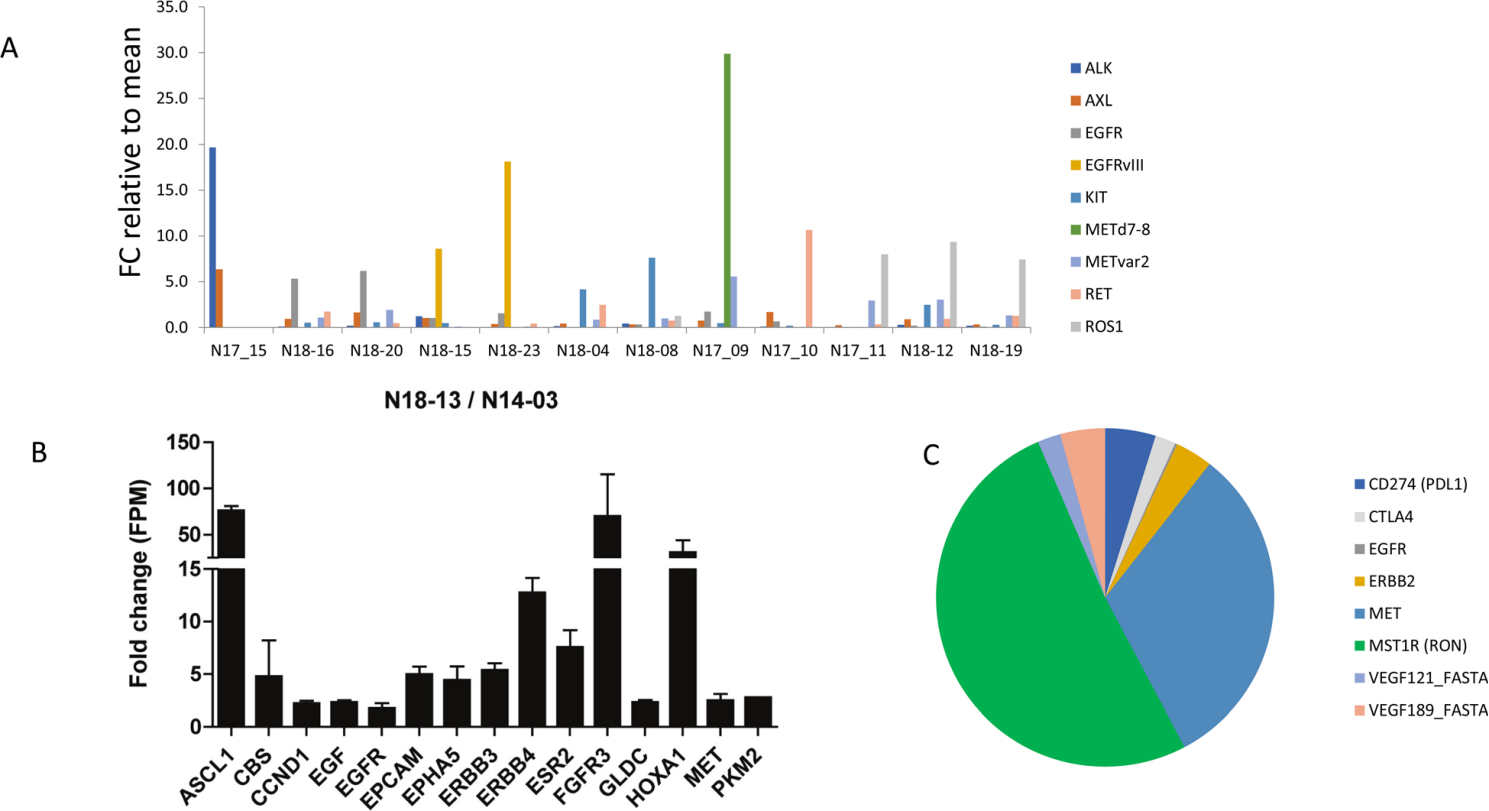
Actionable markers in brain tumours. **a** overview of expression of potentially actionable targets in a subset of patients, expressed as fold change compared to the mean of the entire group. For clarity only tyrosine kinases are shown for a small selection of patients. **b** comparative analysis of a primary pleomorphous xantoastrocytoma and its recurrence 4 years later. **c** shows a selection of actionable targets in sample 13–05, a metastasis of a lung cancer. Note high expression levels of the macrophage receptor RON, MET and CTLA-4. Also VEGF isoforms 121 and 189 are detected

We then investigated whether t/RNA-NGS can stratify patient tumours based on relatively high expression of actionable targets (arbitrarily defined as > 2 fold expression compared to the mean of all gliomas). Results of a selection of tyrosine kinases (EGFR, MET, ALK, AXL, KIT, RET, ROS1) for 12 gliomas are presented in Fig. 6a. In only one tumour high expression levels of CD2, CD3, CD4, CD8 CTLA-4 and PD-1 were found, suggesting an inflammatory phenotype (not shown). Many mutations were detected in the assay that are described as somatic mutations in the Cosmic database. Potentially interesting mutations that we found (based on FATHMM score) included p.RET-S1002R, p.EGFR-G719D, p.EGFR-A289D, PTEN mutations ad TP53 mutations (not shown).

In the pleomorphic xanthoastrocyoma (PXA) in our test cohort a *BRAF*^*V600E*^ mutation was found which was confirmed via genetic testing (Table 1). This mutation has been described before in PXA [46]. These are mostly slow growing, low-grade gliomas but some patients will undergo malignant progression. Mutations in BRAF have been identified as poor prognostic factors for this tumour type [47, 48]. Interestingly, patient 14–03 was operated for a recurrent tumour 4 years later (sample 18–13 in the validation cohort). Samples 14–03 and 18–13 were therefore re-analyzed with t/RNA-NGS in the same sequencing run. The recurrent tumour showed a 70–80 fold increase of expression of transcription factors ASCL1 and HOXA1 and tyrosine kinases FGFR3, HER3, HER4 and MET (Fig. 6b). Another interesting finding was high expression of estrogen receptor 2 (ESR2) in the recurrent, but not the primary tumour. Whereas the p.BRAF-V600E mutation was retained, the recurrent tumour showed an additional p.NF1-E1571* nonsense mutation (not shown). This mutation has been described as pathogenic in the Cosmic database (https://cancer.sanger.ac.uk/cosmic/mutation/overview?id=133079) (FATHMM score = 0.99).

Other observations that were of potential clinical relevance were that the brain metastasis of lung carcinoma expressed relatively high levels of MET, RON and CTLA-4 (Fig. 7C). This may suggest sensitivity to the MET/RON inhibitor BMS777607/ASLAN002, combined with immune checkpoint inhibition [49]. The ependymoma showed high levels of EGFR^vIII^ (not shown). One glioblastoma expressed the MET^∆7–8^ variant that we described previously [31]. In the DNET, considered as a benign grade I tumour, we found high expression levels of ErbB3, FGFR2, FOLH1 and AXL.

### Validation cohort

To validate these results, we next performed t/RNA-NGS on an independent validation cohort of 26 analyzable glioma samples. p.IDH1-R132H mutations were found in 7 samples, all diagnosed as grade II/III astrocytomas and oligodendrogliomas (Table 1). Differentially expressed genes between IDH^mut^ and IDH^wt^ gliomas in the training set were confirmed in the validation cohort (Additional file 6: Table S5). EGFR^vIII^ expression was found in 4 of 19 (21%) of IDHwt glioblastomas and not in p.IDH1-R132H cancers, confirming results in the test cohort. Thus, the test is robust and yields batch-independent clinically relevant information. Survival data in the validation set could not be interpreted due to short time of follow-up.

## Discussion

We here describe the clinical application of a relatively novel and cost-effective multiplex next generation RNA sequencing assay for cancer pathway profiling. The assay generates expression profiles and sequence information of genes that have been identified by literature surveys as potentially important for diagnosis, prognosis and response to precision medicines in a variety of cancer types. We previously demonstrated that the test allows analysis of metabolism by measuring relative expression levels of metabolic enzymes [36, 38]. To analyze clinically actionable pathways we expanded the assay with smMIPs to quantify expression levels of and detect mutations in the involved genes. Analysis of the cohort of 103 brain cancers, of which 97 well characterized gliomas, allowed robust group-based analysis, and confirmed high inter tumour molecular heterogeneity with respect to tyrosine kinase expression. Indeed, the biology in individual gliomas that drives progression is mostly a black box. Therefore, in the routine clinical setting molecular genetics is increasingly implemented as add-on to histopathology to aid the diagnostic process, but influence on therapeutic decision making is limited. Biomarkers that influence clinical management include *IDH* mutations, combined with loss of chromosome arms 1p and 19q, leading to a diagnosis of oligodendroglioma that responds well to PCV, whereas methylation of the *MGMT* promoter in glioblastoma predicts a better response to TMZ [50–52]. *EGFR* status is also determined, amplification of which, in combination with the EGFR^vIII^ mutation, is associated with glioblastoma [53]. We here show that a single t/RNA-NGS test gives the same genetic information that can be derived from DNA analysis, but additionally provides information on gene activity from mutated but also non-mutated alleles. Thus, RNA-profiling is better suited than DNA-profiling to draw conclusions on activity of biological pathways, and thus may be more reliable as a guide to personalized treatment plans [54, 55]. It must be noted that for this study we isolated RNA from tumour tissue, snap-frozen directly after surgery. Although t/RNA-NGS can also be performed on RNA isolated from FFPE tissue, we found that whereas the quality of t/RNA-NGS data is good if tissue is formalin-fixed directly after surgery, it decreases in case of large time lags between surgery and formalin fixation (data not shown).

Tumours with overexpression of a targetable tyrosine kinase will be resistant to the corresponding inhibitor if other kinases are present that converge on the same signaling pathways [56]. Insight in the entire repertoire of expressed tyrosine kinases, downstream signaling intermediates and counteracting phosphatases may therefore predict intrinsic resistance or rapid development thereof [57–59]. t/RNA-NGS provides such insight for individual glioma patients, and even can identify gene activities that have so far been associated with other cancer types, opening new avenues for targeted therapy. For example, expression of the prostate cancer marker PSMA on microvasculature of glioblastoma as we show here, may provide opportunities for tumour-vascular targeting [60–62]. Of interest, it was reported before that high expression levels of AR in glioblastoma translates into sensitivity to the AR antagonist enzalutamide [63]. Notably, our data also confirmed the identification of CA12v1 as a marker of poor prognosis in glioblastoma patients [64]. CA12 is a transmembrane enzyme that is involved in intracellular pH homeostasis and extracellular acidification, providing a biological explanation for the association with poor survival [65]. Overexpression of CA12v1, but not CA12v2, in cell models leads to acidification of the extracellular milieu, whereas overexpression of C12v2 leads to increased oxygen consumption (manuscript in preparation). Because CA12 is expressed at low levels in normal brain, it is a interesting therapeutic target for the group of CA12-positive glioblastomas [66].

In conclusion we here present a novel t/RNA-NGS ‘one size fits all’ assay that can provide a molecular diagnosis and prognosis for glioma and other cancers, as well as generate a list of (actionable) gene expression levels and mutations for individual cancers. The work described in this manuscript was performed with RNA, isolated from optimally biobanked tissue. The small size of the RNA regions of interest (150 bases) that is required for successful smMIP capture, however also allows testing of RNA isolated from formalin fixed, paraffin embedded tissue blocks, provided that autolysis is prevented by proper and rapid processing. The low costs and high throughput capacity of the assay (up to 400 samples can be processed simultaneously due to barcoding) makes it highly interesting to further analyze biobanked tissues in future studies and identify classifier profiles that can predict prognosis and response to therapies. Thus, t/RNA-NGS may allow repurposing of drugs in an individualized manner.

## Supplementary information

**Additional file 1: Figure S1.** Kaplan-Meier analysis of unsupervised clusters of the entire cohort. Indicated in the figure are (a) ependymoma, (b) lung metastasis, (c) DNET, (d) LPD, and (e) variant glioma.

**Additional file 2: Table S1a.** Differential gene expression in cluster **A vs. B.** A total of 83 genes were differentially expressed between the two clusters. Only genes that were significantly different are shown. Mean gene expression values (FPM) values for the clusters are given. For significance: A Wilcoxon-Mann-Whitney test with multiple testing correction was performed. Values are significant when the *p*-value is lower than the False Discovery Rate (FDR). The cutoff for the FDR was < 0.05. **Table S1b**: Differential gene expression in cluster B vs. C. A total of 69 genes were differentially expressed between the two clusters. Only genes that were significantly different are shown. Mean gene expression values (FPM) values for the clusters are given. For significance: A Wilcoxon-Mann-Whitney test with multiple testing correction was performed. Values are significant when the p-value is lower than the False Discovery Rate (FDR). The cutoff for the FDR was < 0.05. **Table S1c**: Differential gene expression in cluster A vs. C. A total of 9 genes were differentially expressed between the two clusters. Only genes that were significantly different are shown. Mean gene expression values (FPM) values for the clusters are given. For significance: A Wilcoxon-Mann-Whitney test with multiple testing correction was performed. Values are significant when the *p*-value is lower than the False Discovery Rate (FDR). The cutoff for the FDR was < 0.05.

**Additional file 3: Table S2.** Mutation detection in glioma samples. A Fisher’s exact test was performed to identify genetic mutations that distinguish the clusters A, B, and C as defined in Fig. 1. Mutations that were present in at least 10% of the unique reads were tested for significance. There were no significant differences between cluster A vs. C. The cutoff for the FDR was set at 0.01. Genes shown here were significantly different between clusters.

**Additional file 4: Table S3.** Differential gene expression in IDH^IDHwt^ gliomas from cluster B vs cluster A**.** A total of 4 genes were differentially expressed between the cluster A and the IDH^IDHwt^ gliomas that grouped in cluster B. Only genes that were significantly different are shown. Mean gene expression values (FPM) values for the clusters are given. For significance: A Wilcoxon-Mann-Whitney test with multiple testing correction was performed. Values are significant when the *p*-value is lower than the False Discovery Rate (FDR). The cutoff for the FDR was < 0.05.

**Additional file 5: Table S4a.** Differential gene expression in oligodendroglioma (O) vs. glioblastoma (G). A total of 79 genes were differentially expressed between the two histological types. Only genes that were significantly different are shown. Mean gene expression values (FPM) values for the histological type are given. For significance: A Wilcoxon-Mann-Whitney test with multiple testing correction was performed. Values are significant when the *p*-value is lower than the False Discovery Rate (FDR). The cutoff for the FDR was < 0.05. **Table S4b.** Differential gene expression in Astrocytomas (A) vs. glioblastoma (G). A total of 50 genes were differentially expressed between the two histological types. Only genes that were significantly different are shown. Mean gene expression values (FPM) values for the histological type are given. For significance: A Wilcoxon-Mann-Whitney test with multiple testing correction was performed. Values are significant when the p-value is lower than the False Discovery Rate (FDR). The cutoff for the FDR was < 0.05. **Table S4c.** Differential gene expression in Astrocytomas (A) vs. oligodendroglioma (O). One gene was differentially expressed between the two histological types. Only genes that were significantly different are shown. Mean gene expression values (FPM) values for the histological type are given. For significance: A Wilcoxon-Mann-Whitney test with multiple testing correction was performed. Values are significant when the p-value is lower than the False Discovery Rate (FDR). The cutoff for the FDR was < 0.05.

**Additional file 6: Table S5.** Analysis of validation cohort. Shown are fold changes of gene expression values, calculated by dividing mean values of each transcript in the group of IDHwt tumors by that in the group of IDHmut tumors in the test cohort and validation cohort.

## References

[1] Basu S, Murphy ME (2016). Genetic modifiers of the p53 pathway. Cold Spring Harb Perspect Med.

[2] Baugh EH, Ke H, Levine AJ, Bonneau RA, Chan CS (2018). Why are there hotspot mutations in the TP53 gene in human cancers?. Cell Death Differ.

[3] Bhattacharya P, Patel TN (2018). Microsatellite instability and promoter Hypermethylation of DNA repair genes in hematologic malignancies: a forthcoming direction toward diagnostics. Hematology.

[4] Du Z, Lovly CM (2018). Mechanisms of receptor tyrosine kinase activation in cancer. Mol Cancer.

[5] Hendriks Wiljan, Bourgonje Annika, Leenders William, Pulido Rafael (2018). Proteinaceous Regulators and Inhibitors of Protein Tyrosine Phosphatases. Molecules.

[6] Choudhry H, Harris AL (2018). Advances in hypoxia-inducible factor biology. Cell Metab.

[7] Petrova V, Annicchiarico-Petruzzelli M, Melino G, Amelio I (2018). The hypoxic tumour microenvironment. Oncogenesis.

[8] Potente M, Gerhardt H, Carmeliet P (2011). Basic and therapeutic aspects of angiogenesis. Cell.

[9] Ghosh D, Nandi S, Bhattacharjee S (2018). Combination therapy to checkmate Glioblastoma: clinical challenges and advances. Clin Transl Med.

[10] Ariazi EA, Jordan VC (2006). Estrogen-related receptors as emerging targets in cancer and metabolic disorders. Curr Top Med Chem.

[11] Gaillard-Moguilewsky M (1991). Pharmacology of antiandrogens and value of combining androgen suppression with antiandrogen therapy. Urology.

[12] Lenting K, Verhaak R, Ter Laan M, Wesseling P, Leenders W (2017). Glioma: experimental models and reality. Acta Neuropathol.

[13] Pant S, Hubbard J, Martinelli E, Bekaii-Saab T (2018). Clinical update on K-Ras targeted therapy in gastrointestinal cancers. Crit Rev Oncol Hematol.

[14] Agianian B, Gavathiotis E (2018). Current insights of BRAF inhibitors in Cancer. J Med Chem.

[15] Mondesir J, Willekens C, Touat M, de Botton S (2016). IDH1 and IDH2 mutations as novel therapeutic targets: current perspectives. J Blood Med.

[16] Nicolaides T. P., Li H., Solomon D. A., Hariono S., Hashizume R., Barkovich K., Baker S. J., Paugh B. S., Jones C., Forshew T., Hindley G. F., Hodgson J. G., Kim J.-S., Rowitch D. H., Weiss W. A., Waldman T. A., James C. D. (2011). Targeted Therapy for BRAFV600E Malignant Astrocytoma. Clinical Cancer Research.

[17] Rubin Mark A, Demichelis Francesca (2018). The Genomics of Prostate Cancer: emerging understanding with technologic advances. Modern Pathology.

[18] Eijkelenboom A, Kamping EJ, Kastner-van Raaij AW, Hendriks-Cornelissen SJ, Neveling K, Kuiper RP, Hoischen A, Nelen MR, Ligtenberg MJ, Tops BB (2016). Reliable next-generation sequencing of formalin-fixed, paraffin-embedded tissue using single molecule tags. J Mol Diagn.

[19] Gorgannezhad L, Umer M, Islam MN, Nguyen NT, Shiddiky MJA (2018). Circulating tumor DNA and liquid biopsy: opportunities, challenges, and recent advances in detection technologies. Lab Chip.

[20] Hyman David M., Piha-Paul Sarina A., Won Helen, Rodon Jordi, Saura Cristina, Shapiro Geoffrey I., Juric Dejan, Quinn David I., Moreno Victor, Doger Bernard, Mayer Ingrid A., Boni Valentina, Calvo Emiliano, Loi Sherene, Lockhart Albert C., Erinjeri Joseph P., Scaltriti Maurizio, Ulaner Gary A., Patel Juber, Tang Jiabin, Beer Hannah, Selcuklu S. Duygu, Hanrahan Aphrothiti J., Bouvier Nancy, Melcer Myra, Murali Rajmohan, Schram Alison M., Smyth Lillian M., Jhaveri Komal, Li Bob T., Drilon Alexander, Harding James J., Iyer Gopa, Taylor Barry S., Berger Michael F., Cutler Jr Richard E., Xu Feng, Butturini Anna, Eli Lisa D., Mann Grace, Farrell Cynthia, Lalani Alshad S., Bryce Richard P., Arteaga Carlos L., Meric-Bernstam Funda, Baselga José, Solit David B. (2018). HER kinase inhibition in patients with HER2- and HER3-mutant cancers. Nature.

[21] Neveling Kornelia, Mensenkamp Arjen R., Derks Ronny, Kwint Michael, Ouchene Hicham, Steehouwer Marloes, van Lier Bart, Bosgoed Ermanno, Rikken Alwin, Tychon Marloes, Zafeiropoulou Dimitra, Castelein Steven, Hehir-Kwa Jayne, Tjwan Thung Djie, Hofste Tom, Lelieveld Stefan H., Bertens Stijn M.M., Adan Ivo B.J.F., Eijkelenboom Astrid, Tops Bastiaan B., Yntema Helger, Stokowy Tomasz, Knappskog Per M., Høberg-Vetti Hildegunn, Steen Vidar M., Boyle Evan, Martin Beth, Ligtenberg Marjolijn J.L., Shendure Jay, Nelen Marcel R., Hoischen Alexander (2016). BRCA Testing by Single-Molecule Molecular Inversion Probes. Clinical Chemistry.

[22] Brien GL, Valerio DG, Armstrong SA (2016). Exploiting the Epigenome to control Cancer-promoting gene-expression programs. Cancer Cell.

[23] Capper David, Jones David T. W., Sill Martin, Hovestadt Volker, Schrimpf Daniel, Sturm Dominik, Koelsche Christian, Sahm Felix, Chavez Lukas, Reuss David E., Kratz Annekathrin, Wefers Annika K., Huang Kristin, Pajtler Kristian W., Schweizer Leonille, Stichel Damian, Olar Adriana, Engel Nils W., Lindenberg Kerstin, Harter Patrick N., Braczynski Anne K., Plate Karl H., Dohmen Hildegard, Garvalov Boyan K., Coras Roland, Hölsken Annett, Hewer Ekkehard, Bewerunge-Hudler Melanie, Schick Matthias, Fischer Roger, Beschorner Rudi, Schittenhelm Jens, Staszewski Ori, Wani Khalida, Varlet Pascale, Pages Melanie, Temming Petra, Lohmann Dietmar, Selt Florian, Witt Hendrik, Milde Till, Witt Olaf, Aronica Eleonora, Giangaspero Felice, Rushing Elisabeth, Scheurlen Wolfram, Geisenberger Christoph, Rodriguez Fausto J., Becker Albert, Preusser Matthias, Haberler Christine, Bjerkvig Rolf, Cryan Jane, Farrell Michael, Deckert Martina, Hench Jürgen, Frank Stephan, Serrano Jonathan, Kannan Kasthuri, Tsirigos Aristotelis, Brück Wolfgang, Hofer Silvia, Brehmer Stefanie, Seiz-Rosenhagen Marcel, Hänggi Daniel, Hans Volkmar, Rozsnoki Stephanie, Hansford Jordan R., Kohlhof Patricia, Kristensen Bjarne W., Lechner Matt, Lopes Beatriz, Mawrin Christian, Ketter Ralf, Kulozik Andreas, Khatib Ziad, Heppner Frank, Koch Arend, Jouvet Anne, Keohane Catherine, Mühleisen Helmut, Mueller Wolf, Pohl Ute, Prinz Marco, Benner Axel, Zapatka Marc, Gottardo Nicholas G., Driever Pablo Hernáiz, Kramm Christof M., Müller Hermann L., Rutkowski Stefan, von Hoff Katja, Frühwald Michael C., Gnekow Astrid, Fleischhack Gudrun, Tippelt Stephan, Calaminus Gabriele, Monoranu Camelia-Maria, Perry Arie, Jones Chris, Jacques Thomas S., Radlwimmer Bernhard, Gessi Marco, Pietsch Torsten, Schramm Johannes, Schackert Gabriele, Westphal Manfred, Reifenberger Guido, Wesseling Pieter, Weller Michael, Collins Vincent Peter, Blümcke Ingmar, Bendszus Martin, Debus Jürgen, Huang Annie, Jabado Nada, Northcott Paul A., Paulus Werner, Gajjar Amar, Robinson Giles W., Taylor Michael D., Jaunmuktane Zane, Ryzhova Marina, Platten Michael, Unterberg Andreas, Wick Wolfgang, Karajannis Matthias A., Mittelbronn Michel, Acker Till, Hartmann Christian, Aldape Kenneth, Schüller Ulrich, Buslei Rolf, Lichter Peter, Kool Marcel, Herold-Mende Christel, Ellison David W., Hasselblatt Martin, Snuderl Matija, Brandner Sebastian, Korshunov Andrey, von Deimling Andreas, Pfister Stefan M. (2018). DNA methylation-based classification of central nervous system tumours. Nature.

[24] Capper David, Stichel Damian, Sahm Felix, Jones David T. W., Schrimpf Daniel, Sill Martin, Schmid Simone, Hovestadt Volker, Reuss David E., Koelsche Christian, Reinhardt Annekathrin, Wefers Annika K., Huang Kristin, Sievers Philipp, Ebrahimi Azadeh, Schöler Anne, Teichmann Daniel, Koch Arend, Hänggi Daniel, Unterberg Andreas, Platten Michael, Wick Wolfgang, Witt Olaf, Milde Till, Korshunov Andrey, Pfister Stefan M., von Deimling Andreas (2018). Practical implementation of DNA methylation and copy-number-based CNS tumor diagnostics: the Heidelberg experience. Acta Neuropathologica.

[25] Kangsamaksin T, Tattersall IW, Kitajewski J (2014). Notch functions in developmental and tumour angiogenesis by diverse mechanisms. Biochem Soc Trans.

[26] Saharinen P, Eklund L, Pulkki K, Bono P, Alitalo K (2011). VEGF and angiopoietin signaling in tumor angiogenesis and metastasis. Trends Mol Med.

[27] Kusters B, de Waal RM, Wesseling P, Verrijp K, Maass C, Heerschap A, Barentsz JO, Sweep F, Ruiter DJ, Leenders WP (2003). Differential effects of vascular endothelial growth factor a isoforms in a mouse brain metastasis model of human melanoma. Cancer Res.

[28] Frampton G. M., Ali S. M., Rosenzweig M., Chmielecki J., Lu X., Bauer T. M., Akimov M., Bufill J. A., Lee C., Jentz D., Hoover R., Ou S.-H. I., Salgia R., Brennan T., Chalmers Z. R., Jaeger S., Huang A., Elvin J. A., Erlich R., Fichtenholtz A., Gowen K. A., Greenbowe J., Johnson A., Khaira D., McMahon C., Sanford E. M., Roels S., White J., Greshock J., Schlegel R., Lipson D., Yelensky R., Morosini D., Ross J. S., Collisson E., Peters M., Stephens P. J., Miller V. A. (2015). Activation of MET via Diverse Exon 14 Splicing Alterations Occurs in Multiple Tumor Types and Confers Clinical Sensitivity to MET Inhibitors. Cancer Discovery.

[29] Greenall SA, Johns TG (2016). EGFRvIII: the promiscuous mutation. Cell Death Discov.

[30] Lowenstein PR, Castro MG (2014) The value of EGFRvIII as the target for glioma vaccines. AM Soc Clin Oncol Educ book: 42-50 Doi 10.14694/EdBook_AM.2014.34.42 10.14694/EdBook_AM.2014.34.42 PMC4438702 24857059

[31] Navis Anna C., van Lith Sanne A. M., van Duijnhoven Sander M. J., de Pooter Maaike, Yetkin-Arik Bahar, Wesseling Pieter, Hendriks Wiljan J. A. J., Venselaar Hanka, Timmer Marco, van Cleef Patricia, van Bergen en Henegouwen Paul, Best Myron G., Wurdinger Thomas D., Tops Bastiaan B. J., Leenders William P. J. (2015). Identification of a novel MET mutation in high-grade glioma resulting in an auto-active intracellular protein. Acta Neuropathologica.

[32] Sottoriva A, Spiteri I, Piccirillo SG, Touloumis A, Collins VP, Marioni JC, Curtis C, Watts C, Tavare S (2013). Intratumor heterogeneity in human glioblastoma reflects cancer evolutionary dynamics. Proc Natl Acad Sci U S A.

[33] Stupp Roger, Mason Warren P., van den Bent Martin J., Weller Michael, Fisher Barbara, Taphoorn Martin J.B., Belanger Karl, Brandes Alba A., Marosi Christine, Bogdahn Ulrich, Curschmann Jürgen, Janzer Robert C., Ludwin Samuel K., Gorlia Thierry, Allgeier Anouk, Lacombe Denis, Cairncross J. Gregory, Eisenhauer Elizabeth, Mirimanoff René O. (2005). Radiotherapy plus Concomitant and Adjuvant Temozolomide for Glioblastoma. New England Journal of Medicine.

[34] Claes A, Idema AJ, Wesseling P (2007). Diffuse glioma growth: a guerilla war. Acta Neuropathol.

[35] de Bitter T, van de Water C, van den Heuvel C, Zeelen C, Eijkelenboom A, Tops B, Oosterwijk E, Kolev D, Mulders P, Ter Laan Met al (2017) Profiling of the metabolic transcriptome via single molecule molecular inversion probes. Sci Rep 7: 11402 Doi 10.1038/s41598-017-11035-0 10.1038/s41598-017-11035-0 PMC5595890 28900252

[36] Lenting Krissie, Khurshed Mohammed, Peeters Tom H., van den Heuvel Corina N. A. M., van Lith Sanne A. M., de Bitter Tessa, Hendriks Wiljan, Span Paul N., Molenaar Remco J., Botman Dennis, Verrijp Kiek, Heerschap Arend, ter Laan Mark, Kusters Benno, van Ewijk Anne, Huynen Martijn A., van Noorden Cornelis J. F., Leenders William P. J. (2019). Isocitrate dehydrogenase 1–mutated human gliomas depend on lactate and glutamate to alleviate metabolic stress. The FASEB Journal.

[37] van den Heuvel C, Das AI, de Bitter T, Simmer F, Wurdinger T, Molina-Vila MA, Leenders WPJ (2018). Quantification and localization of oncogenic receptor tyrosine kinase variant transcripts using molecular inversion probes. Sci Rep.

[38] van den Heuvel C, van Ewijk A, Zeelen C, de Bitter T, Huynen M, Mulders P, Oosterwijk E, Leenders WPJ (2019). Molecular profiling of Druggable targets in clear cell renal cell carcinoma through targeted RNA sequencing. Front Oncol.

[39] Lenting Krissie, Khurshed Mohammed, Peeters Tom H., van den Heuvel Corina N. A. M., van Lith Sanne A. M., de Bitter Tessa, Hendriks Wiljan, Span Paul N., Molenaar Remco J., Botman Dennis, Verrijp Kiek, Heerschap Arend, ter Laan Mark, Kusters Benno, van Ewijk Anne, Huynen Martijn A., van Noorden Cornelis J. F., Leenders William P. J. (2019). Isocitrate dehydrogenase 1–mutated human gliomas depend on lactate and glutamate to alleviate metabolic stress. The FASEB Journal.

[40] O'Roak B. J., Vives L., Fu W., Egertson J. D., Stanaway I. B., Phelps I. G., Carvill G., Kumar A., Lee C., Ankenman K., Munson J., Hiatt J. B., Turner E. H., Levy R., O'Day D. R., Krumm N., Coe B. P., Martin B. K., Borenstein E., Nickerson D. A., Mefford H. C., Doherty D., Akey J. M., Bernier R., Eichler E. E., Shendure J. (2012). Multiplex Targeted Sequencing Identifies Recurrently Mutated Genes in Autism Spectrum Disorders. Science.

[41] Galili T (2015). Dendextend: an R package for visualizing, adjusting and comparing trees of hierarchical clustering. Bioinformatics.

[42] Dobin A, Davis CA, Schlesinger F, Drenkow J, Zaleski C, Jha S, Batut P, Chaisson M, Gingeras TR (2013). STAR: ultrafast universal RNA-seq aligner. Bioinformatics.

[43] Louis DN, Perry A, Reifenberger G, von Deimling A, Figarella-Branger D, Cavenee WK, Ohgaki H, Wiestler OD, Kleihues P, Ellison DW (2016). The 2016 World Health Organization classification of tumors of the central nervous system: a summary. Acta Neuropathol.

[44] Tönjes Martje, Barbus Sebastian, Park Yoon Jung, Wang Wei, Schlotter Magdalena, Lindroth Anders M, Pleier Sabrina V, Bai Alfa H C, Karra Daniela, Piro Rosario M, Felsberg Jörg, Addington Adele, Lemke Dieter, Weibrecht Irene, Hovestadt Volker, Rolli Claudio G, Campos Benito, Turcan Sevin, Sturm Dominik, Witt Hendrik, Chan Timothy A, Herold-Mende Christel, Kemkemer Ralf, König Rainer, Schmidt Kathrin, Hull William-Edmund, Pfister Stefan M, Jugold Manfred, Hutson Susan M, Plass Christoph, Okun Jürgen G, Reifenberger Guido, Lichter Peter, Radlwimmer Bernhard (2013). BCAT1 promotes cell proliferation through amino acid catabolism in gliomas carrying wild-type IDH1. Nature Medicine.

[45] Gan HK, Kaye AH, Luwor RB (2009). The EGFRvIII variant in glioblastoma multiforme. J Clin Neurosci.

[46] Stone Thomas J., Keeley Angus, Virasami Alex, Harkness William, Tisdall Martin, Izquierdo Delgado Elisa, Gutteridge Alice, Brooks Tony, Kristiansen Mark, Chalker Jane, Wilkhu Lisa, Mifsud William, Apps John, Thom Maria, Hubank Mike, Forshew Tim, Cross J. Helen, Hargrave Darren, Ham Jonathan, Jacques Thomas S. (2017). Comprehensive molecular characterisation of epilepsy-associated glioneuronal tumours. Acta Neuropathologica.

[47] Dahiya S, Haydon DH, Alvarado D, Gurnett CA, Gutmann DH, Leonard JR (2013). BRAF(V600E) mutation is a negative prognosticator in pediatric ganglioglioma. Acta Neuropathol.

[48] Wang Junmei, Liu Zhaoxia, Cui Yun, Liu Yuqing, Fang Jingyi, Xu Li, He Yanjiao, Du Jiang, Su Yujin, Zou Wanjing, Xu Zuolin, Li Guilin (2019). Evaluation of EZH2 expression, BRAF V600E mutation, and CDKN2A/B deletions in epithelioid glioblastoma and anaplastic pleomorphic xanthoastrocytoma. Journal of Neuro-Oncology.

[49] Ekiz HA, Lai SA, Gundlapalli H, Haroun F, Williams MA, Welm AL (2018). Inhibition of RON kinase potentiates anti-CTLA-4 immunotherapy to shrink breast tumors and prevent metastatic outgrowth. Oncoimmunology.

[50] Chen Y, Hu F, Zhou Y, Chen W, Shao H, Zhang Y (2013). MGMT promoter methylation and glioblastoma prognosis: a systematic review and meta-analysis. Arch Med Res.

[51] Hegi ME, Stupp R (2015). Withholding temozolomide in glioblastoma patients with unmethylated MGMT promoter--still a dilemma?. Neuro-Oncology.

[52] Molenaar Remco J., Verbaan Dagmar, Lamba Simona, Zanon Carlo, Jeuken Judith W.M., Boots-Sprenger Sandra H.E., Wesseling Pieter, Hulsebos Theo J.M., Troost Dirk, van Tilborg Angela A., Leenstra Sieger, Vandertop W. Peter, Bardelli Alberto, van Noorden Cornelis J.F., Bleeker Fonnet E. (2014). The combination of IDH1 mutations and MGMT methylation status predicts survival in glioblastoma better than either IDH1 or MGMT alone. Neuro-Oncology.

[53] Phillips A. C., Boghaert E. R., Vaidya K. S., Mitten M. J., Norvell S., Falls H. D., DeVries P. J., Cheng D., Meulbroek J. A., Buchanan F. G., McKay L. M., Goodwin N. C., Reilly E. B. (2016). ABT-414, an Antibody-Drug Conjugate Targeting a Tumor-Selective EGFR Epitope. Molecular Cancer Therapeutics.

[54] Eckhardt S. Gail, Lieu Christopher (2019). Is Precision Medicine an Oxymoron?. JAMA Oncology.

[55] Trédan O, Wang Q, Pissaloux D, Cassier P, de la Fouchardière A, Fayette J, Desseigne F, Ray-Coquard I, de la Fouchardière C, Frappaz D, Heudel P -E, Bonneville-Levard A, Fléchon A, Sarabi M, Guibert P, Bachelot T, Pérol M, You B, Bonnin N, Collard O, Leyronnas C, Attignon V, Baudet C, Sohier E, Villemin J -P, Viari A, Boyault S, Lantuejoul S, Paindavoine S, Treillleux I, Rodriguez C, Agrapart V, Corset V, Garin G, Chabaud S, Pérol D, Blay J -Y (2019). Molecular screening program to select molecular-based recommended therapies for metastatic cancer patients: analysis from the ProfiLER trial. Annals of Oncology.

[56] van den Heuvel Corina N.A.M., Navis Anna C., de Bitter Tessa, Amiri Houshang, Verrijp Kiek, Heerschap Arend, Rex Karen, Dussault Isabelle, Caenepeel Sean, Coxon Angela, Span Paul N., Wesseling Pieter, Hendriks Wiljan, Leenders William P.J. (2017). Selective MET Kinase Inhibition in MET-Dependent Glioma Models Alters Gene Expression and Induces Tumor Plasticity. Molecular Cancer Research.

[57] Birkman EM, Elzagheid A, Jokilehto T, Avoranta T, Korkeila E, Kulmala J, Syrjanen K, Westermarck J, Sundstrom J (2018). Protein phosphatase 2A (PP2A) inhibitor CIP2A indicates resistance to radiotherapy in rectal cancer. Cancer Med.

[58] Navis AC, Bourgonje A, Wesseling P, Wright A, Hendriks W, Verrijp K, van der Laak JA, Heerschap A, Leenders WP (2013). Effects of dual targeting of tumor cells and stroma in human glioblastoma xenografts with a tyrosine kinase inhibitor against c-MET and VEGFR2. PLoS One.

[59] van den Heuvel Corina N.A.M., Navis Anna C., de Bitter Tessa, Amiri Houshang, Verrijp Kiek, Heerschap Arend, Rex Karen, Dussault Isabelle, Caenepeel Sean, Coxon Angela, Span Paul N., Wesseling Pieter, Hendriks Wiljan, Leenders William P.J. (2017). Selective MET Kinase Inhibition in MET-Dependent Glioma Models Alters Gene Expression and Induces Tumor Plasticity. Molecular Cancer Research.

[60] Nomura Natsuko, Pastorino Sandra, Jiang Pengfei, Lambert Gage, Crawford John R, Gymnopoulos Marco, Piccioni David, Juarez Tiffany, Pingle Sandeep C, Makale Milan, Kesari Santosh (2014). Prostate specific membrane antigen (PSMA) expression in primary gliomas and breast cancer brain metastases. Cancer Cell International.

[61] Salas Fragomeni RA, Menke JR, Holdhoff M, Ferrigno C, Laterra JJ, Solnes LB, Javadi MS, Szabo Z, Pomper MG, Rowe SP (2017). Prostate-specific membrane antigen-targeted imaging with [18F] DCFPyL in high-grade Gliomas. Clin Nucl Med.

[62] Wernicke AG, Edgar MA, Lavi E, Liu H, Salerno P, Bander NH, Gutin PH (2011). Prostate-specific membrane antigen as a potential novel vascular target for treatment of glioblastoma multiforme. Arch Pathol Lab Med.

[63] Zalcman N, Canello T, Ovadia H, Charbit H, Zelikovitch B, Mordechai A, Fellig Y, Rabani S, Shahar T, Lossos Aet al (2018) Androgen receptor: a potential therapeutic target for glioblastoma. Oncotarget 9: 19980-19993 Doi 10.18632/oncotarget.25007 10.18632/oncotarget.25007 PMC5929440 29731997

[64] Haapasalo Joonas, Hilvo Mika, Nordfors Kristiina, Haapasalo Hannu, Parkkila Seppo, Hyrskyluoto Alise, Rantala Immo, Waheed Abdul, Sly William S., Pastorekova Silvia, Pastorek Jaromir, Parkkila Anna-Kaisa (2008). Identification of an alternatively spliced isoform of carbonic anhydrase XII in diffusely infiltrating astrocytic gliomas. Neuro-Oncology.

[65] Mboge Mam, Mahon Brian, McKenna Robert, Frost Susan (2018). Carbonic Anhydrases: Role in pH Control and Cancer. Metabolites.

[66] Fiedler L., Kellner M., Gosewisch A., Oos R., Böning G., Lindner S., Albert N., Bartenstein P., Reulen H.-J., Zeidler R., Gildehaus F.J. (2018). Evaluation of 177Lu[Lu]-CHX-A″-DTPA-6A10 Fab as a radioimmunotherapy agent targeting carbonic anhydrase XII. Nuclear Medicine and Biology.

