# Supplementary material for: Mapping actionable pathways and mutations in brain tumours using targeted RNA next generation sequencing
Source: Acta Neuropathol Commun. 2019 Nov 20;7:185. doi: 10.1186/s40478-019-0826-z (PMC6865071; doi:10.1186/s40478-019-0826-z)
Supplement: Supplementary file 1 — Additional file 1: Figure S1. Kaplan-Meier analysis of unsupervised clusters of the entire cohort. Indicated in the figure are (a) ependymoma, (b) lung metastasis, (c) DNET, (d) LPD, and (e) variant glioma. [file 40478_2019_826_MOESM1_ESM.docx]

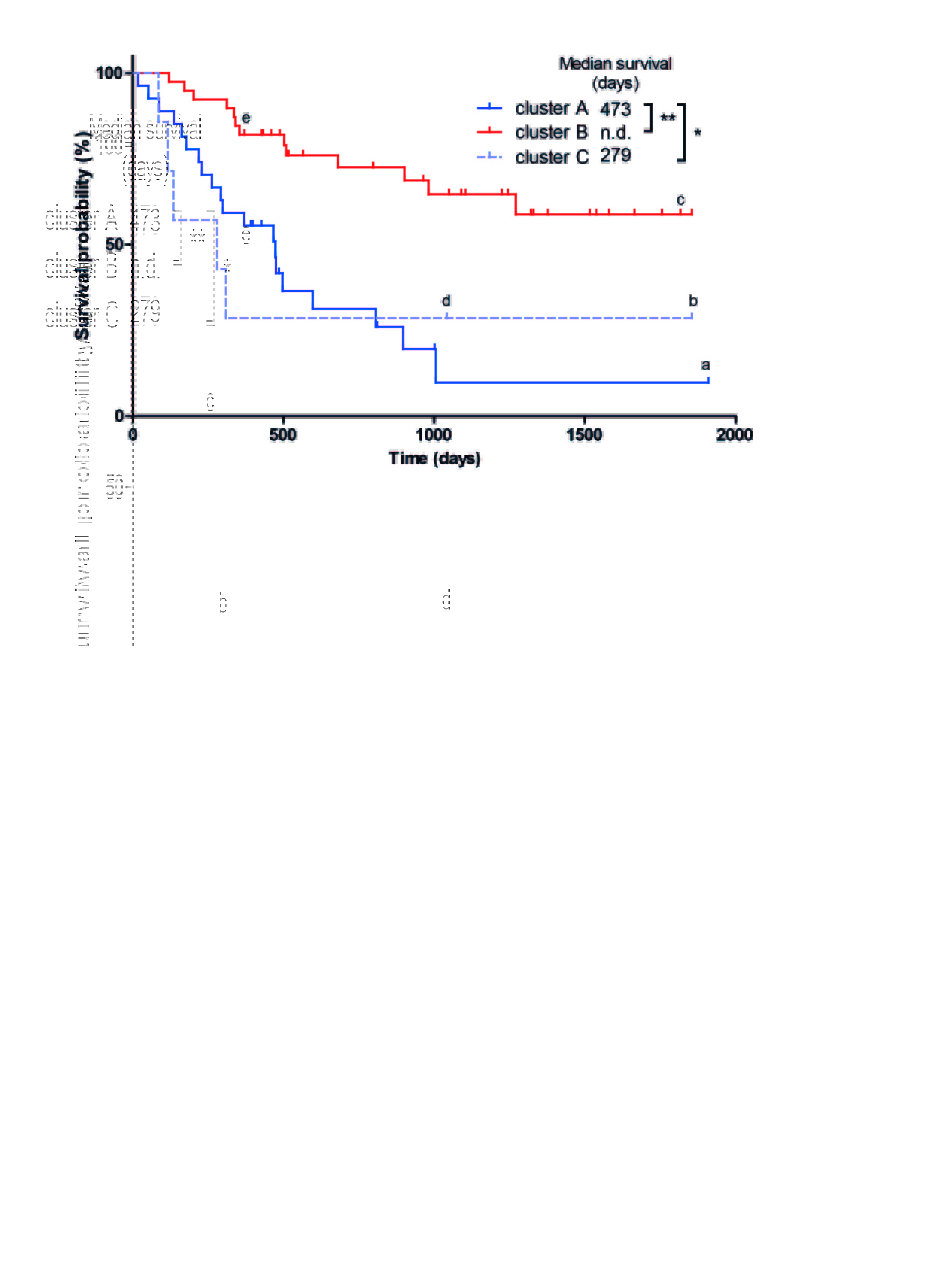
**Figure S1: Kaplan-Meier analysis of unsupervised clusters of the entire cohort.** Indicated in the figure are (a) ependymoma, (b) lung metastasis, (c) DNET, (d) LPD, and (e) variant glioma.
