# Supplementary material for: Mapping actionable pathways and mutations in brain tumours using targeted RNA next generation sequencing
Source: Acta Neuropathol Commun. 2019 Nov 20;7:185. doi: 10.1186/s40478-019-0826-z (PMC6865071; doi:10.1186/s40478-019-0826-z)
Supplement: Supplementary file 2 — Additional file 2: Table S1a. Differential gene expression in cluster A vs. B. A total of 83 genes were differentially expressed between the two clusters. Only genes that were significantly different are shown. Mean gene expression values (FPM) values for the clusters are given. For significance: A Wilcoxon-Mann-Whitney test with multiple testing correction was performed. Values are significant when the p-value is lower than the False Discovery Rate (FDR). The cutoff for the FDR was < 0.05. Table S1b: Differential gene expression in cluster B vs. C. A total of 69 genes were differentially expressed between the two clusters. Only genes that were significantly different are shown. Mean gene expression values (FPM) values for the clusters are given. For significance: A Wilcoxon-Mann-Whitney test with multiple testing correction was performed. Values are significant when the p-value is lower than the False Discovery Rate (FDR). The cutoff for the FDR was < 0.05. Table S1c: Differential gene expression in cluster A vs. C. A total of 9 genes were differentially expressed between the two clusters. Only genes that were significantly different are shown. Mean gene expression values (FPM) values for the clusters are given. For significance: A Wilcoxon-Mann-Whitney test with multiple testing correction was performed. Values are significant when the p-value is lower than the False Discovery Rate (FDR). The cutoff for the FDR was < 0.05. [file 40478_2019_826_MOESM2_ESM.docx]

**Table SIa: Differential gene expression in cluster A vs. B.** A total of 83 genes were differentially expressed between the two clusters. Only genes that were significantly different are shown. Mean gene expression values (FPM) values for the clusters are given. For significance: A Wilcoxon-Mann-Whitney test with multiple testing correction was performed. Values are significant when the p-value is lower than the False Discovery Rate (FDR). The cutoff for the FDR was <0.05.

| Gene | Mean FPM Cluster A | Mean FPM Cluster B | p-value | FDR |
| --- | --- | --- | --- | --- |
| VEGF | 315.843 | 18.989 | 0.000 | 0.000 |
| VEGF165 | 1650.128 | 91.928 | 0.000 | 0.000 |
| VEGF121 | 865.636 | 80.282 | 0.000 | 0.000 |
| LDHA | 4376.568 | 939.698 | 0.000 | 0.000 |
| BCAT1 | 1605.473 | 244.772 | 0.000 | 0.000 |
| PFKM | 460.133 | 1206.420 | 0.000 | 0.000 |
| ERBB4 | 62.190 | 326.643 | 0.000 | 0.000 |
| ABAT | 359.512 | 1282.089 | 0.000 | 0.001 |
| GLUD1 | 839.99f3 | 4044.100 | 0.000 | 0.001 |
| GLUD2 | 35.906 | 225.705 | 0.000 | 0.001 |
| ATP5C1 | 1144.432 | 2469.982 | 0.000 | 0.001 |
| VEGF189 | 997.068 | 40.234 | 0.000 | 0.001 |
| LDHB | 3783.220 | 7991.827 | 0.000 | 0.001 |
| SLC16A3 | 454.556 | 131.831 | 0.000 | 0.001 |
| MAPK8 | 117.256 | 295.313 | 0.000 | 0.001 |
| NTRK2 | 4462.448 | 15774.166 | 0.000 | 0.001 |
| GCLC | 229.046 | 687.386 | 0.000 | 0.001 |
| GAD1 | 94.355 | 557.142 | 0.000 | 0.001 |
| CA12 | 1303.110 | 172.401 | 0.000 | 0.001 |
| PGAM1 | 428.430 | 762.698 | 0.000 | 0.001 |
| ACO2 | 202.578 | 380.995 | 0.000 | 0.001 |
| ACACA | 231.076 | 416.369 | 0.000 | 0.002 |
| CBS | 131.479 | 381.506 | 0.000 | 0.002 |
| CA9 | 92.920 | 13.781 | 0.000 | 0.002 |
| PC | 135.112 | 336.137 | 0.000 | 0.002 |
| IDH3G | 330.658 | 556.237 | 0.000 | 0.002 |
| SDHA | 647.412 | 1055.310 | 0.000 | 0.002 |
| CS | 402.670 | 695.179 | 0.000 | 0.002 |
| NAMPT | 5014.847 | 883.489 | 0.000 | 0.002 |
| PDK1 | 361.496 | 165.003 | 0.000 | 0.002 |
| PGK1 | 3663.771 | 1848.398 | 0.000 | 0.002 |
| GPI_1 | 749.152 | 444.488 | 0.000 | 0.002 |
| IDH3A | 145.390 | 249.574 | 0.000 | 0.002 |
| GOT1 | 42.813 | 90.941 | 0.000 | 0.002 |
| SOD1 | 410.087 | 758.264 | 0.000 | 0.002 |
| HK2 | 65.428 | 18.863 | 0.000 | 0.002 |
| ATP5A1 | 2196.228 | 3204.543 | 0.000 | 0.003 |
| GAPDH | 33982.269 | 24284.599 | 0.000 | 0.003 |
| GLUL | 1640.171 | 3617.271 | 0.000 | 0.003 |
| SLC2A3 | 4293.117 | 1573.138 | 0.000 | 0.003 |
| MDH1 | 349.529 | 572.232 | 0.000 | 0.003 |
| L2HGDH | 20.233 | 37.599 | 0.000 | 0.003 |
| GPI_2 | 1076.352 | 644.286 | 0.000 | 0.003 |
| CHKA | 98.455 | 167.233 | 0.000 | 0.003 |
| BRAF | 244.947 | 377.284 | 0.000 | 0.003 |
| D2HGDH | 81.096 | 132.612 | 0.000 | 0.003 |
| SOD2 | 20373.181 | 8933.506 | 0.000 | 0.003 |
| IGF1R | 459.821 | 566.188 | 0.000 | 0.003 |
| MERTK | 339.525 | 816.026 | 0.000 | 0.003 |
| ERBB3 | 67.403 | 261.207 | 0.000 | 0.003 |
| PDHA1 | 275.098 | 461.988 | 0.000 | 0.004 |
| SLC7A1 | 983.578 | 1586.329 | 0.000 | 0.004 |
| HK3 | 11.139 | 4.010 | 0.000 | 0.004 |
| GPT | 3.435 | 7.871 | 0.000 | 0.004 |
| NOX4 | 103.698 | 62.475 | 0.000 | 0.004 |
| ENO1 | 12011.763 | 8521.523 | 0.000 | 0.004 |
| GCLM | 159.109 | 99.892 | 0.000 | 0.004 |
| PDGFRB | 548.986 | 315.916 | 0.000 | 0.004 |
| SDHC | 899.070 | 1188.473 | 0.000 | 0.004 |
| SLC9A1 | 336.077 | 215.883 | 0.000 | 0.004 |
| TXN | 2489.500 | 1721.999 | 0.000 | 0.004 |
| ARHGAP26 | 715.163 | 1194.567 | 0.000 | 0.004 |
| IDH2 | 504.126 | 827.137 | 0.000 | 0.004 |
| SLC1A2 | 3676.856 | 9430.761 | 0.000 | 0.004 |
| FASN | 132.675 | 231.527 | 0.000 | 0.004 |
| PDGFRA | 5031.468 | 5126.429 | 0.000 | 0.005 |
| PLXND1 | 275.485 | 191.349 | 0.000 | 0.005 |
| PTEN | 538.754 | 858.624 | 0.000 | 0.005 |
| FBP1 | 47.942 | 28.475 | 0.000 | 0.005 |
| CKB | 3661.729 | 6620.678 | 0.000 | 0.005 |
| EGFRvIII | 253.316 | 1.001 | 0.001 | 0.005 |
| MST1R | 0.845 | 1.551 | 0.001 | 0.005 |
| SDHD | 625.821 | 818.604 | 0.001 | 0.005 |
| CPT1A | 256.524 | 358.950 | 0.001 | 0.005 |
| MET | 52.833 | 34.974 | 0.001 | 0.005 |
| IDH1 | 1172.620 | 780.466 | 0.001 | 0.005 |
| TALDO1 | 1145.332 | 1631.526 | 0.002 | 0.005 |
| EGLN1 | 189.116 | 247.098 | 0.002 | 0.005 |
| PRKAA2 | 32.568 | 48.941 | 0.002 | 0.005 |
| PKM | 4859.248 | 3775.165 | 0.003 | 0.006 |
| NAPRT1 | 6.831 | 13.359 | 0.003 | 0.006 |
| ACSS2 | 131.275 | 178.672 | 0.004 | 0.006 |
| KIT | 213.857 | 206.096 | 0.004 | 0.006 |

**Table SIb: Differential gene expression in cluster B vs. C.** A total of 69 genes were differentially expressed between the two clusters. Only genes that were significantly different are shown. Mean gene expression values (FPM) values for the clusters are given. For significance: A Wilcoxon-Mann-Whitney test with multiple testing correction was performed. Values are significant when the p-value is lower than the False Discovery Rate (FDR). The cutoff for the FDR was <0.05.

| Gene | Mean FPM cluster B | Mean FPM cluster C | p-value | FDR |
| --- | --- | --- | --- | --- |
| ABAT | 1282.089 | 33.210 | 0.000 | 0.000 |
| ALK | 77.621 | 3.386 | 0.000 | 0.000 |
| ATP5C1 | 2469.982 | 752.070 | 0.000 | 0.000 |
| CBS | 381.506 | 35.986 | 0.000 | 0.000 |
| CHKA | 167.233 | 45.718 | 0.000 | 0.000 |
| ERBB3 | 261.207 | 3.718 | 0.000 | 0.000 |
| ERBB4 | 326.643 | 2.236 | 0.000 | 0.000 |
| GLUD1 | 4044.100 | 313.730 | 0.000 | 0.001 |
| GLUD2 | 225.705 | 11.736 | 0.000 | 0.001 |
| LDHB | 7991.827 | 1867.272 | 0.000 | 0.001 |
| NTRK2 | 15774.166 | 754.240 | 0.000 | 0.001 |
| PC | 336.137 | 45.400 | 0.000 | 0.001 |
| SLC16A3 | 131.831 | 1405.726 | 0.000 | 0.001 |
| GLDC | 369.237 | 35.338 | 0.000 | 0.001 |
| KIT | 206.096 | 10.122 | 0.000 | 0.001 |
| L2HGDH | 37.599 | 7.534 | 0.000 | 0.001 |
| VEGF121 | 80.282 | 1057.350 | 0.000 | 0.001 |
| CKB | 6620.678 | 486.208 | 0.000 | 0.001 |
| GAD1 | 557.142 | 14.752 | 0.000 | 0.001 |
| HK2 | 18.863 | 241.470 | 0.000 | 0.001 |
| PFKM | 1206.420 | 219.006 | 0.000 | 0.001 |
| VEGF | 18.989 | 513.152 | 0.000 | 0.002 |
| ACO2 | 380.995 | 102.326 | 0.000 | 0.002 |
| D2HGDH | 132.612 | 40.816 | 0.000 | 0.002 |
| ENO1 | 8521.523 | 19276.616 | 0.000 | 0.002 |
| MAPK8 | 295.313 | 74.022 | 0.000 | 0.002 |
| SDHC | 1188.473 | 482.868 | 0.000 | 0.002 |
| SLC2A3 | 1573.138 | 10808.804 | 0.000 | 0.002 |
| CS | 695.179 | 253.322 | 0.000 | 0.002 |
| IDH2 | 827.137 | 221.878 | 0.000 | 0.002 |
| LDHA | 939.698 | 5143.006 | 0.000 | 0.002 |
| SLC1A2 | 9430.761 | 369.618 | 0.000 | 0.002 |
| SLC7A1 | 1586.329 | 409.818 | 0.000 | 0.002 |
| PGK1 | 1848.398 | 5752.602 | 0.000 | 0.002 |
| BCAT1 | 244.772 | 1294.698 | 0.000 | 0.002 |
| NAMPT | 883.489 | 10081.262 | 0.000 | 0.002 |
| MDH1 | 572.232 | 230.760 | 0.000 | 0.003 |
| RET | 21.796 | 0.986 | 0.000 | 0.003 |
| IDH3A | 249.574 | 85.394 | 0.000 | 0.003 |
| IDH3G | 556.237 | 256.586 | 0.000 | 0.003 |
| VEGF165 | 91.928 | 746.332 | 0.000 | 0.003 |
| ACSS2 | 178.672 | 73.680 | 0.000 | 0.003 |
| FGFR2 | 189.059 | 16.696 | 0.000 | 0.003 |
| GCLC | 687.386 | 150.580 | 0.000 | 0.003 |
| PTEN | 858.624 | 281.650 | 0.000 | 0.003 |
| BRAF | 377.284 | 148.594 | 0.000 | 0.003 |
| PDHA1 | 461.988 | 163.292 | 0.000 | 0.003 |
| SDHD | 818.604 | 437.902 | 0.000 | 0.003 |
| CAT | 359.956 | 158.610 | 0.001 | 0.003 |
| EGFR | 233.221 | 18.110 | 0.001 | 0.003 |
| PARP1 | 1327.669 | 543.882 | 0.001 | 0.004 |
| PKM | 3775.165 | 6456.298 | 0.001 | 0.004 |
| VEGF189 | 40.234 | 934.224 | 0.001 | 0.004 |
| ALDOA | 9682.345 | 14013.498 | 0.001 | 0.004 |
| ATP5A1 | 3204.543 | 1366.026 | 0.001 | 0.004 |
| EGLN1 | 247.098 | 120.326 | 0.001 | 0.004 |
| CA9 | 13.781 | 223.078 | 0.001 | 0.004 |
| GOT1 | 90.941 | 36.028 | 0.001 | 0.004 |
| SOD1 | 758.264 | 348.312 | 0.001 | 0.004 |
| PRKAA2 | 48.941 | 16.718 | 0.001 | 0.004 |
| VHL1 | 237.804 | 89.154 | 0.002 | 0.004 |
| HK3 | 4.010 | 34.536 | 0.002 | 0.004 |
| FH | 209.338 | 101.728 | 0.002 | 0.004 |
| ACACA | 416.369 | 156.574 | 0.002 | 0.004 |
| PFKFB1 | 2.239 | 0.544 | 0.004 | 0.004 |
| AXL | 374.522 | 134.300 | 0.004 | 0.005 |
| MET | 34.974 | 105.468 | 0.004 | 0.005 |
| KDR | 556.674 | 76.816 | 0.005 | 0.005 |
| PDGFRA | 5126.429 | 1009.234 | 0.005 | 0.005 |

**Table SIc: Differential gene expression in cluster A vs. C.** A total of 9 genes were differentially expressed between the two clusters. Only genes that were significantly different are shown. Mean gene expression values (FPM) values for the clusters are given. For significance: A Wilcoxon-Mann-Whitney test with multiple testing correction was performed. Values are significant when the p-value is lower than the False Discovery Rate (FDR). The cutoff for the FDR was <0.05.

| Gene | mean FMP cluster A | Mean FPM cluster C | p-value | FDR |
| --- | --- | --- | --- | --- |
| ABAT | 359.5123077 | 33.21 | 0.000 | 0.000 |
| GLUD1 | 839.9930769 | 313.73 | 0.000 | 0.000 |
| ERBB4 | 62.19 | 2.236 | 0.000 | 0.000 |
| GLDC | 276.0173077 | 35.338 | 0.000 | 0.000 |
| KDR | 388.2826923 | 76.816 | 0.000 | 0.000 |
| FOLH1 | 473.1838462 | 30.216 | 0.000 | 0.000 |
| FGFR2 | 129.5269231 | 16.696 | 0.000 | 0.000 |
| GLUD2 | 35.90576923 | 11.736 | 0.000 | 0.000 |
| NOX4 | 103.6980769 | 24.384 | 0.000 | 0.000 |
