# Supplementary material for: Mapping actionable pathways and mutations in brain tumours using targeted RNA next generation sequencing
Source: Acta Neuropathol Commun. 2019 Nov 20;7:185. doi: 10.1186/s40478-019-0826-z (PMC6865071; doi:10.1186/s40478-019-0826-z)
Supplement: Supplementary file 3 — Additional file 3: Table S2. Mutation detection in glioma samples. A Fisher’s exact test was performed to identify genetic mutations that distinguish the clusters A, B, and C as defined in Fig. 1. Mutations that were present in at least 10% of the unique reads were tested for significance. There were no significant differences between cluster A vs. C. The cutoff for the FDR was set at 0.01. Genes shown here were significantly different between clusters. [file 40478_2019_826_MOESM3_ESM.docx]

**Supplementary table SII: Mutation detection in glioma samples.** A Fisher’s exact test was performed to identify genetic mutations that distinguish the clusters A, B, and C as defined in Figure 1. Mutations that were present in at least 10 percent of the unique reads were tested for significance. There were no significant differences between cluster A vs. C. The cutoff for the FDR was set at 0.01. Genes shown here were significantly different between clusters.

| Cluster A vs. B |  |  |  |  |  |
| --- | --- | --- | --- | --- | --- |
| Gene mutation | **Gene mutation** | **Present in A** | **Present in B** | **p-value** | **FDR** |
| IDH1 | p.Arg132His | 0/26 | 28/38 | 4.44E-10 | 3.11E-05 |
|  |  |  |  |  |  |
| Cluster B vs. C |  |  |  |  |  |
| Gene mutation | **Gene mutation** | **Present in B** | **Present in C** | **p-value** | **FDR** |
| SDHA | p.Leu18_Ala19dup | 37/38 | 0/5 | 6.23E-06 | 2.98E-05 |
