# Supplementary material for: Mapping actionable pathways and mutations in brain tumours using targeted RNA next generation sequencing
Source: Acta Neuropathol Commun. 2019 Nov 20;7:185. doi: 10.1186/s40478-019-0826-z (PMC6865071; doi:10.1186/s40478-019-0826-z)
Supplement: Supplementary file 4 — Additional file 4: Table S3. Differential gene expression in IDHIDHwt gliomas from cluster B vs cluster A. A total of 4 genes were differentially expressed between the cluster A and the IDHIDHwt gliomas that grouped in cluster B. Only genes that were significantly different are shown. Mean gene expression values (FPM) values for the clusters are given. For significance: A Wilcoxon-Mann-Whitney test with multiple testing correction was performed. Values are significant when the p-value is lower than the False Discovery Rate (FDR). The cutoff for the FDR was < 0.05. [file 40478_2019_826_MOESM4_ESM.docx]

**Supplementary table SIV: Differential gene expression in IDH^IDHwt^ gliomas from cluster B vs cluster A.** A total of 4 genes were differentially expressed between the cluster A and the IDH^IDHwt^ gliomas that grouped in cluster B. Only genes that were significantly different are shown. Mean gene expression values (FPM) values for the clusters are given. For significance: A Wilcoxon-Mann-Whitney test with multiple testing correction was performed. Values are significant when the p-value is lower than the False Discovery Rate (FDR). The cutoff for the FDR was <0.05.

| Gene | Mean FPM cluster A | mean FPM IDH^IDHWT^s in cluster B | p-value | FDR |
| --- | --- | --- | --- | --- |
| VEGF165 | 1666.451 | 58.500 | 0.000 | 0.000 |
| VEGF189 | 977.172 | 41.042 | 0.000 | 0.000 |
| VEGF | 316.292 | 13.778 | 0.000 | 0.000 |
| VEGF121 | 865.596 | 65.298 | 0.000 | 0.000 |
