# Supplementary material for: Mapping actionable pathways and mutations in brain tumours using targeted RNA next generation sequencing
Source: Acta Neuropathol Commun. 2019 Nov 20;7:185. doi: 10.1186/s40478-019-0826-z (PMC6865071; doi:10.1186/s40478-019-0826-z)
Supplement: Supplementary file 5 — Additional file 5: Table S4a. Differential gene expression in oligodendroglioma (O) vs. glioblastoma (G). A total of 79 genes were differentially expressed between the two histological types. Only genes that were significantly different are shown. Mean gene expression values (FPM) values for the histological type are given. For significance: A Wilcoxon-Mann-Whitney test with multiple testing correction was performed. Values are significant when the p-value is lower than the False Discovery Rate (FDR). The cutoff for the FDR was < 0.05. Table S4b. Differential gene expression in Astrocytomas (A) vs. glioblastoma (G). A total of 50 genes were differentially expressed between the two histological types. Only genes that were significantly different are shown. Mean gene expression values (FPM) values for the histological type are given. For significance: A Wilcoxon-Mann-Whitney test with multiple testing correction was performed. Values are significant when the p-value is lower than the False Discovery Rate (FDR). The cutoff for the FDR was < 0.05. Table S4c. Differential gene expression in Astrocytomas (A) vs. oligodendroglioma (O). One gene was differentially expressed between the two histological types. Only genes that were significantly different are shown. Mean gene expression values (FPM) values for the histological type are given. For significance: A Wilcoxon-Mann-Whitney test with multiple testing correction was performed. Values are significant when the p-value is lower than the False Discovery Rate (FDR). The cutoff for the FDR was < 0.05. [file 40478_2019_826_MOESM5_ESM.docx]

**Table SIVa: Differential gene expression in oligodendroglioma (O) vs. glioblastoma (G).** A total of 79 genes were differentially expressed between the two histological types. Only genes that were significantly different are shown. Mean gene expression values (FPM) values for the histological type are given. For significance: A Wilcoxon-Mann-Whitney test with multiple testing correction was performed. Values are significant when the p-value is lower than the False Discovery Rate (FDR). The cutoff for the FDR was <0.05.

| Gene | Mean FPM O | Mean FPM G | p-value | FDR |
| --- | --- | --- | --- | --- |
| LDHA | 463.02 | 4028.73 | 0.000 | 0.000 |
| PFKM | 1407.65 | 500.74 | 0.000 | 0.000 |
| ABAT | 1583.34 | 399.70 | 0.000 | 0.000 |
| BCAT1 | 144.99 | 1381.35 | 0.000 | 0.000 |
| LDHB | 8884.68 | 4012.27 | 0.000 | 0.000 |
| CBS | 402.38 | 137.79 | 0.000 | 0.000 |
| CS | 736.33 | 404.79 | 0.000 | 0.000 |
| SLC16A3 | 89.69 | 531.71 | 0.000 | 0.001 |
| ACACA | 472.83 | 242.18 | 0.000 | 0.001 |
| GLUD1 | 4025.21 | 975.93 | 0.000 | 0.001 |
| SOD2 | 5038.11 | 19897.21 | 0.000 | 0.001 |
| GAD1 | 722.64 | 143.80 | 0.000 | 0.001 |
| GPI_1 | 381.53 | 708.30 | 0.000 | 0.001 |
| GLUD2 | 218.58 | 46.31 | 0.000 | 0.001 |
| ERBB4 | 318.29 | 84.29 | 0.000 | 0.001 |
| NAMPT | 596.93 | 4997.24 | 0.000 | 0.001 |
| PC | 366.68 | 137.33 | 0.000 | 0.001 |
| NTRK2 | 14968.00 | 5092.23 | 0.000 | 0.001 |
| PGAM1 | 892.07 | 477.72 | 0.000 | 0.001 |
| ATP5C1 | 2483.77 | 1222.98 | 0.000 | 0.001 |
| SOD1 | 898.90 | 438.60 | 0.000 | 0.001 |
| IDH3G | 608.65 | 343.61 | 0.000 | 0.002 |
| SDHA | 1184.38 | 704.97 | 0.000 | 0.002 |
| VEGF121 | 88.85 | 742.05 | 0.000 | 0.002 |
| HK2 | 13.10 | 81.33 | 0.000 | 0.002 |
| VEGF | 23.96 | 285.94 | 0.000 | 0.002 |
| GOT1 | 106.80 | 48.45 | 0.000 | 0.002 |
| MAPK8 | 317.58 | 133.45 | 0.000 | 0.002 |
| GPI_2 | 530.35 | 990.43 | 0.000 | 0.002 |
| IGF1R | 575.63 | 399.28 | 0.000 | 0.002 |
| VEGF189 | 46.73 | 811.61 | 0.000 | 0.002 |
| ACO2 | 409.97 | 210.37 | 0.000 | 0.002 |
| GCLC | 732.70 | 269.77 | 0.000 | 0.002 |
| VEGF165 | 118.02 | 1236.81 | 0.000 | 0.002 |
| IDH3A | 277.82 | 154.24 | 0.000 | 0.002 |
| D2HGDH | 151.50 | 80.91 | 0.000 | 0.002 |
| SLC7A1 | 1706.62 | 934.94 | 0.000 | 0.003 |
| SLC2A3 | 1301.77 | 4686.36 | 0.000 | 0.003 |
| SLC9A1 | 170.53 | 346.93 | 0.000 | 0.003 |
| L2HGDH | 40.92 | 20.11 | 0.000 | 0.003 |
| ATP5A1 | 3468.10 | 2187.43 | 0.000 | 0.003 |
| SDHC | 1285.31 | 880.65 | 0.000 | 0.003 |
| PDHA1 | 521.22 | 279.67 | 0.000 | 0.003 |
| CA9 | 17.94 | 93.48 | 0.000 | 0.003 |
| MDH1 | 603.55 | 369.90 | 0.000 | 0.003 |
| MET | 10.84 | 55.95 | 0.000 | 0.003 |
| PGK1 | 1967.94 | 3595.70 | 0.000 | 0.003 |
| FBP1 | 17.48 | 59.52 | 0.000 | 0.003 |
| ALK | 128.78 | 47.28 | 0.000 | 0.003 |
| CHKA | 177.20 | 106.08 | 0.000 | 0.003 |
| CKB | 7498.28 | 3500.81 | 0.000 | 0.004 |
| BRAF | 402.55 | 252.92 | 0.000 | 0.004 |
| SLC16A7 | 176.87 | 136.02 | 0.000 | 0.004 |
| SLC16A1 | 681.52 | 1137.67 | 0.000 | 0.004 |
| CA12 | 227.88 | 1055.67 | 0.000 | 0.004 |
| TALDO1 | 1782.59 | 1193.87 | 0.000 | 0.004 |
| PDGFRA | 5294.95 | 4427.43 | 0.000 | 0.004 |
| ENO1 | 8027.94 | 12392.53 | 0.000 | 0.004 |
| SDHD | 868.54 | 628.45 | 0.000 | 0.004 |
| ERBB3 | 349.27 | 78.66 | 0.000 | 0.004 |
| EGLN1 | 274.98 | 185.77 | 0.000 | 0.004 |
| FH | 228.96 | 171.13 | 0.000 | 0.004 |
| GAPDH | 25070.21 | 32132.06 | 0.000 | 0.004 |
| PDK1 | 179.88 | 312.18 | 0.000 | 0.004 |
| FASN | 242.72 | 158.25 | 0.001 | 0.004 |
| HK3 | 4.04 | 12.87 | 0.001 | 0.005 |
| CPT1A | 410.11 | 276.92 | 0.001 | 0.005 |
| GCLM | 92.92 | 146.69 | 0.001 | 0.005 |
| PFKFB1 | 2.55 | 1.51 | 0.001 | 0.005 |
| GPT | 8.25 | 3.66 | 0.001 | 0.005 |
| G6PC | 1.83 | 0.33 | 0.002 | 0.005 |
| IDH2 | 854.75 | 520.60 | 0.002 | 0.005 |
| GLUL | 3973.52 | 1858.98 | 0.002 | 0.005 |
| PLXND1 | 173.71 | 336.24 | 0.002 | 0.005 |
| CD274 | 13.98 | 27.86 | 0.002 | 0.005 |
| ADPGK | 196.23 | 308.57 | 0.003 | 0.005 |
| SLC1A2 | 9679.14 | 5159.91 | 0.003 | 0.005 |
| PTEN | 855.24 | 551.86 | 0.004 | 0.005 |
| GLDC | 409.85 | 247.87 | 0.005 | 0.005 |

**Table SIVb: Differential gene expression in Astrocytomas (A) vs. glioblastoma (G).** A total of 50 genes were differentially expressed between the two histological types. Only genes that were significantly different are shown. Mean gene expression values (FPM) values for the histological type are given. For significance: A Wilcoxon-Mann-Whitney test with multiple testing correction was performed. Values are significant when the p-value is lower than the False Discovery Rate (FDR). The cutoff for the FDR was <0.05.

| Gene | Mean FPM A | Mean FPM G | p-value | FDR |
| --- | --- | --- | --- | --- |
| GLUD1 | 5293.31 | 975.93 | 0.000 | 0.000 |
| GLUD2 | 304.69 | 46.31 | 0.000 | 0.000 |
| BCAT1 | 189.25 | 1381.35 | 0.000 | 0.000 |
| NTRK2 | 20109.69 | 5092.23 | 0.000 | 0.000 |
| ATP5C1 | 2809.18 | 1222.98 | 0.000 | 0.000 |
| CA12 | 82.87 | 1055.67 | 0.000 | 0.000 |
| LDHA | 1110.43 | 4028.73 | 0.000 | 0.000 |
| PRKAA2 | 67.07 | 31.50 | 0.000 | 0.001 |
| VEGF189 | 32.90 | 811.61 | 0.000 | 0.001 |
| ERBB4 | 399.15 | 84.29 | 0.000 | 0.001 |
| PFKM | 1094.09 | 500.74 | 0.000 | 0.001 |
| VEGF | 14.87 | 285.94 | 0.000 | 0.001 |
| MAPK8 | 294.62 | 133.45 | 0.000 | 0.001 |
| GLUL | 3749.54 | 1858.98 | 0.000 | 0.001 |
| ABAT | 1080.06 | 399.70 | 0.000 | 0.001 |
| GCLC | 721.35 | 269.77 | 0.000 | 0.001 |
| PGK1 | 1686.07 | 3595.70 | 0.000 | 0.001 |
| VEGF121 | 79.82 | 742.05 | 0.000 | 0.001 |
| VEGF165 | 73.90 | 1236.81 | 0.000 | 0.001 |
| PC | 360.63 | 137.33 | 0.000 | 0.001 |
| LDHB | 7509.53 | 4012.27 | 0.000 | 0.001 |
| ACO2 | 372.76 | 210.37 | 0.000 | 0.002 |
| IDH3G | 532.97 | 343.61 | 0.000 | 0.002 |
| MYC | 1673.47 | 638.66 | 0.000 | 0.002 |
| ATP5A1 | 3057.38 | 2187.43 | 0.000 | 0.002 |
| CBR1 | 77.69 | 236.58 | 0.000 | 0.002 |
| MERTK | 883.48 | 415.34 | 0.000 | 0.002 |
| IGF1R | 699.41 | 399.28 | 0.000 | 0.002 |
| EPAS1 | 567.49 | 1146.52 | 0.000 | 0.002 |
| CBS | 434.54 | 137.79 | 0.000 | 0.002 |
| SLC16A3 | 162.31 | 531.71 | 0.000 | 0.002 |
| GAPDH | 22245.75 | 32132.06 | 0.000 | 0.002 |
| CS | 731.72 | 404.79 | 0.000 | 0.002 |
| PDK1 | 147.57 | 312.18 | 0.000 | 0.002 |
| CA9 | 13.50 | 93.48 | 0.000 | 0.002 |
| SLC7A1 | 1662.44 | 934.94 | 0.000 | 0.002 |
| L2HGDH | 37.57 | 20.11 | 0.000 | 0.003 |
| GAD1 | 375.31 | 143.80 | 0.000 | 0.003 |
| NAMPT | 1094.00 | 4997.24 | 0.001 | 0.003 |
| ACACA | 368.84 | 242.18 | 0.001 | 0.003 |
| GPI_1 | 462.18 | 708.30 | 0.001 | 0.003 |
| PKM | 3186.93 | 4822.66 | 0.001 | 0.003 |
| ERBB3 | 172.65 | 78.66 | 0.001 | 0.003 |
| D2HGDH | 116.55 | 80.91 | 0.001 | 0.003 |
| GPT | 8.48 | 3.66 | 0.002 | 0.003 |
| PTEN | 901.95 | 551.86 | 0.002 | 0.003 |
| SLC9A1 | 185.68 | 346.93 | 0.002 | 0.003 |
| NQO1 | 69.02 | 171.00 | 0.002 | 0.003 |
| SLC2A3 | 1885.76 | 4686.36 | 0.003 | 0.003 |
| FASN | 256.59 | 158.25 | 0.003 | 0.003 |

**Table SIVc: Differential gene expression in Astrocytomas (A) vs. oligodendroglioma (O).** One gene was differentially expressed between the two histological types. Only genes that were significantly different are shown. Mean gene expression values (FPM) values for the histological type are given. For significance: A Wilcoxon-Mann-Whitney test with multiple testing correction was performed. Values are significant when the p-value is lower than the False Discovery Rate (FDR). The cutoff for the FDR was <0.05.

| Gene | Mean FPM A | Mean FPM O | p-value | FDR |
| --- | --- | --- | --- | --- |
| ALK | 19.61 | 128.78 | 0.000 | 0.000 |
