# Supplementary material for: Mapping actionable pathways and mutations in brain tumours using targeted RNA next generation sequencing
Source: Acta Neuropathol Commun. 2019 Nov 20;7:185. doi: 10.1186/s40478-019-0826-z (PMC6865071; doi:10.1186/s40478-019-0826-z)
Supplement: Supplementary file 6 — Additional file 6: Table S5. Analysis of validation cohort. Shown are fold changes of gene expression values, calculated by dividing mean values of each transcript in the group of IDHwt tumors by that in the group of IDHmut tumors in the test cohort and validation cohort. [file 40478_2019_826_MOESM6_ESM.docx]

Table SV

Analysis of validation cohort. Shown are fold changes of gene expression values, calculated by dividing mean values of each transcript in the group of IDHwt tumors by that in the group of IDHmut tumors in the test cohort and validation cohort.

| ***transcript*** | ***FC wt/mut test set*** | ***FC wt/mut validation set*** |
| --- | --- | --- |
| ABAT | 0.3 | 0.5 |
| ACACA | 0.6 | 0.5 |
| ATP5C1 | 0.5 | 0.6 |
| BCAT1 | 6.6 | 5.1 |
| BRAF | 0.6 | 0.7 |
| CA12 | 7.6 | 5.1 |
| CA9 | 6.7 | 24.1 |
| EGFRvIII | 253.1 | 200.0 |
| EGLN1 | 0.8 | 1.0 |
| ENO1 | 1.4 | 2.0 |
| ERBB3 | 0.3 | 0.7 |
| ERBB4 | 0.2 | 0.3 |
| FASN | 0.6 | 0.6 |
| FBP1 | 1.7 | 1.4 |
| GAD1 | 0.2 | 0.2 |
| GAPDH | 1.4 | 1.3 |
| GCLC | 0.3 | 0.7 |
| GCLM | 1.6 | 1.5 |
| GLUD1 | 0.2 | 0.5 |
| GLUD2 | 0.2 | 0.5 |
| GLUL | 0.5 | 0.8 |
| GOT1 | 0.5 | 0.5 |
| GPI (var3) | 1.7 | 1.3 |
| GPI (var4) | 1.7 | 1.3 |
| GPT | 0.4 | 1.5 |
| HK2 | 3.5 | 6.0 |
| HK3 | 2.8 | 10.4 |
| LDHA | 4.7 | 3.5 |
| LDHB | 0.5 | 0.6 |
| MAPK8 | 0.4 | 0.5 |
| MET | 1.5 | 1.8 |
| MST1R | 0.5 | 0.6 |
| NAMPT | 5.7 | 3.4 |
| NAPRT1 | 0.5 | 2.4 |
| NOX4 | 1.7 | 1.0 |
| NTRK2 | 0.3 | 0.7 |
| PC | 0.4 | 0.9 |
| PDGFRA | 1.0 | 0.5 |
| PDGFRB | 1.7 | 1.8 |
| PDK1 | 2.2 | 3.0 |
| PFKM | 0.4 | 0.7 |
| SLC16A3 | 3.4 | 6.3 |
| SLC1A2 | 0.4 | 0.4 |
| SOD2 | 2.3 | 3.2 |
| VEGFA (full) | 16.6 | 21.1 |
| VEGFA-121 | 10.8 | 10.3 |
| VEGFA-165 | 18.0 | 12.8 |
| VEGFA-189 | 24.8 | 26.6 |
